# Pancreastatin Inhibition Alters the Colonic Epithelial Cells Profile in a Sex-Dependent Manner

**DOI:** 10.3390/ijms252312757

**Published:** 2024-11-27

**Authors:** Diane M. Tshikudi, Hannah Hutchison, Jean-Eric Ghia

**Affiliations:** 1 Department of Immunology, Rady Faculty of Health Sciences, University of Manitoba, Winnipeg, MB R3E 0T5, Canada; tshikudd@myumanitoba.ca (D.M.T.); hutchish@myumanitoba.ca (H.H.); 2 Children’s Hospital Research Institute of Manitoba, University of Manitoba, Winnipeg, MB R3E 3P4, Canada; 3 IBD Clinical and Research Centre, University of Manitoba, Winnipeg, MB R3A 1R9, Canada

**Keywords:** mucosa, pancreastatin, ulcerative colitis

## Abstract

The impaired mucosal barrier is a hallmark of ulcerative colitis (UC), an inflammatory colonic disorder with epidemiological and pathophysiology sex bias. UC Patients overexpress the colonic epithelial cells (CECs)-derived peptide pancreastatin (PST). Pancreastatin inhibitor 8 (PSTi8), an inhibitor of PST, has shown promising anti-inflammatory effects on UC. However, no data exist in the context of CEC barrier function and integrity. We investigated the impact of PSTi8 treatment on CECs in homeostatic and colitic conditions. PSTi8 (2.5 mg/mL/kg, i.r.) or PBS treatment started one day before colitis induction (5% dextran sodium sulfate for five days) in male and female C57BL/6 mice. The disease activity score was assessed daily. Epithelial-associated cytokines, markers specific to differentiation, proliferation, differentiated CECs, stem cells, CECs regulators, and the PSTi8 G-protein coupled receptor 78 (GPR78) signaling pathway, were evaluated using ELISA, immunofluorescence and qRT-PCR. PSTi8 treatment reduced the epithelial-associated cytokines and differentiated CECs while promoting CEC proliferation and self-renewal in females at a steady state through the GRP78 signaling pathway. PSTi8 treatment exacerbated colitis severity and increased CEC differentiation while reducing proliferation in colitic females. Conversely, PSTi8 treatment reduced males’ susceptibility to colitis by preserving stem cells and differentiated CECs. PST regulated colonic mucosal maintenance in a sex- and disease-dependent manner.

## 1. Introduction

The gastrointestinal (GI) mucosa, formed of an epithelium overlaid by a mucus layer, serves as a selectively permeable barrier whose functions are primordial to restrict mucosal infiltration by luminal microorganisms, dietary antigens, and other cellular insults [1]. The GI epithelium regulates the absorption of electrolytes, nutrients, water, and other beneficial molecules while creating a physical, immunological, and chemical barrier between the luminal contents and host immune cells in the lamina propria. The colon epithelium is organized along the crypt axis, with stem cell niches that reside at the base of the crypts and terminally differentiated colonic cells positioned apically from the crypt base [2]. With a relatively short lifespan of 3–5 days, the colonic epithelial cells undergo constant self-renewal mediated by the Leucine-rich repeat-containing G-protein coupled receptor 5 (LGR5^+^) crypt base columnar stem cells (CBCs) to preserve the epithelial barrier homeostasis [3]. In colonic inflammatory pathologies resulting in severe epithelium injury that lead to depletion of Lgr5^+^ cells, damage-associated regenerative cells (DARCs) such as ulcerative colitis (UC), homeodomain-only protein homeobox (HOPX^+^), and fetal-like lymphocyte antigen (LY6A^+^) stem cells can rescue CBCs loss, promote mucosa repair and prevent epithelial barrier dysfunction [1,4]. Epithelial stem cells can give rise to terminally differentiated cells subdivided into absorptive colonocytes and the secretory mucus-secreting goblets, chemosensory Tufts, and hormones-producing enteroendocrine cells [3,5,6]. These terminally differentiated cells maintain the colonic mucosal barrier integrity and function, hence promoting colonic mucosa homeostasis [3]. Interestingly, recent studies reveal that sex hormones such as estrogen, progesterone, and testosterone modulate intestinal barrier function [7,8,9,10]. Therefore, impaired colonic mucosal barrier function can promote the development of GI pathologies such as inflammatory bowel diseases, which include UC [4].

UC is a chronic inflammatory disorder with a higher incidence rate in males than in females [11,12]. Characterized by severe mucosal ulceration along the colon and rectum, UC is associated with periods of remission and relapse symptoms such as bloody diarrhea, abdominal pain, and fecal urgency [13]. Although still under investigation, many studies suggest a central role for compromised gut mucosal barrier function in UC etiopathology [14,15]. Thus, the impaired colonic mucosal barrier in UC is associated with an unresolved endoplasmic reticulum (ER) stress and an unfolded protein response (UPR), a lower number of mature goblets cells, defective mucus production, along with an enhanced epithelial permeability linked to increased expression of pore-forming tight junction (TJ) Claudin 2 and lower expression of regulators of pore-forming TJ Claudin 4 (CLD4) [4,16,17,18]. Moreover, several studies associate UC pathogenesis with altered enteroendocrine cell functions [19,20]. Recent studies revealed that intestinal enteroendocrine cells from UC patients acquire antigen-presenting function through major histocompatibility complex (MHC) class I and MHC II pathways, in addition to undergoing a functional shift that increases their capacity to synthesize proteins and secrete hormones such as serotonin and chromogranin A (CHGA) [19,21]. Ubiquitously distributed in enteroendocrine cells, CHGA can undergo post-translational proteolytic processing and give rise to numerous biological effector molecules, which are known to influence the colonic mucosa integrity and function in homeostatic and pathological conditions [14,22]. One such bioactive peptide, pancreastatin (PST), has been shown to govern glucose homeostasis in the pancreas, skeletal muscle, liver, adipose tissues, and heart, likely through the insulin receptor substrate (IRS)1/2-PI3-kinase-Akt signaling pathway and the (UPR) chaperon molecule glucose-regulated protein of 78 kDa (GRP78) [22,23,24,25,26]. In the GI tract, patients with active UC had significantly higher PST levels than healthy control, which positively correlated with epithelial cell-associated inflammatory cytokines [27]. Moreover, we and others demonstrated that PST supplementation in the CHGA-knockout mice model increases the intestinal mucosal permeability at a steady state, reduces TJ mRNA expression and worsens disease severity in the dextran sodium sulfate (DSS) experimental model of colitis [27,28,29]. However, the influence of PST on colonic epithelial cell functions remains to be investigated. PST inhibitors could help study the effect of PST on the colonic mucosa integrity and functions. So far, pancreastatin variant 1 (PSTv1) and pancreastatin inhibitor 8 (PSTi8), two truncated forms of PST, have been shown as promising competitive inhibitors of PST peripheral metabolic action [25,30]. PSTv1 and PSTi8, N-terminally and C-terminally truncated peptides, could be potential anti-inflammatory mediators in inflammatory-mediated pathologies such as UC [28]. Although several studies report the effect of PST inhibition with PSTv1 and PSTi8 in the context of diet-induced obesity, insulin resistance, and diabetes, studies investigating their impact on inflammatory disorders are limited [28]. While PSTv1-mediated PST inhibition has been found to suppress adipose tissues’ macrophage-mediated inflammation, the mechanism of action by which PSTv1 mediates its inhibitory action is still poorly understood [30]. By contrast, PSTi8, a recently developed anti-diabetic molecule, has been found to compete with PST for binding to the GRP78 receptor active site, attenuates insulin resistance and improves glucose homeostasis while reducing pro-inflammatory cytokine levels [25]. Moreover, studies suggest that PSTi8 treatment alters the IRS1-2-phosphatidylinositol-3-kinase-AKT-Glycogen synthase kinase-3 beta (GSK3β) signaling pathway, known to control cell growth, proliferation, and apoptosis, all of which are known to play a significant role in UC and colitis-associated cancer pathophysiology [31].

Growing evidence suggests that gut mucosal healing in UC patients correlates with an increased risk of hospitalization and surgery [32]. In UC patients, amelioration of gut mucosa healing could improve clinical outcomes and long-term remission for UC patients [14,33]. Therefore, it is crucial to understand factors that influence the mucosal integrity and colonic epithelial barrier function to provide novel molecular targets for UC therapy. In the present study, we explore the potential effects of PST inhibition with PSTi8 on colonic epithelial cell integrity and mucosal barrier functions in male and female mice at a steady state and during DSS-mediated acute colitis.

## 2. Results

### 2.1. PST Inhibition Is Associated with Delayed Colitis Onset in Male Mice but Moderately Worsens Disease Severity in Female Mice

**Figure 1:**
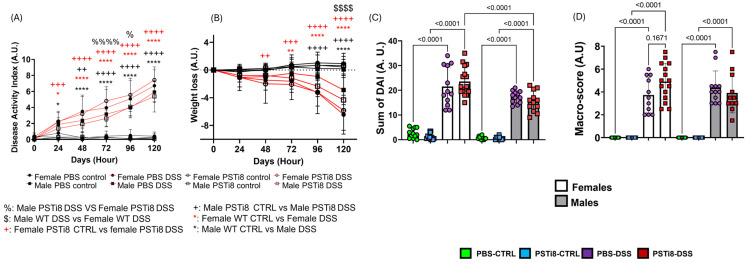
Inhibition of PST delayed colitis onset in males and increased disease severity in female mice. Male and female C57BL/6 (WT) mice (n = 12–15) were administered with PSTi8 (2.5 mg/kg/day) or PBS by intra-rectal route one day before treatment with 5% DSS for five days to induce colitis. The disease activity index score (**A**) and body weight loss (**B**) were calculated daily from stool consistency and bleeding scores. (**C**) The total sum of the DAI score was calculated for each condition. (**D**) The macro score index calculated from rectal bleeding, rectal prolapse, and diarrhea, was evaluated for each mouse on the day of the experiment. Two-way or one-way ANOVA, followed by a multiple comparison test, was performed. The statistical significance of data expressed as the mean ± standard deviation was determined using ordinary one-way ANOVA and Tukey’s multiple comparisons test. Significance signs comparison: * PBS-treated control (CTRL) vs. PBS-treated DSS (DSS). + PSTi8-treated control (PSTi8 CTRL) vs. PSTi8-treated DSS (PSTi8 DSS). $ male PBS-treated DSS vs female PBS-treated DSS. % male PSTi8-treated DSS vs female PSTi8-treated DSS mice. Red significance signs compare conditions between female mice, whereas black signs compare conditions between male mice. *^, %^ *p* < 0.05; **^, ++^ *p* < 0.01; ^+++^ *p* < 0.001; ^****, ++++, %%%%, $$$$^ *p* < 0.0001.

Male and female mice treated with DSS to induce colitis were administrated with PSTi8 to investigate the contribution of PST to colitis severity between sexes. All DSS-treated mice showed a progressive increase in the disease activity index (DAI) score and weight loss along with an elevated sum in DAI score and macro-score compared to non-colitic control mice (*p* < 0.05) (Figure 1A–D). However, colitis severity differed moderately between sexes following treatment with PSTi8. Prophylactic therapy with PSTi8 in males had a trend toward a delayed colitis onset with DAI that shifted from 24 to 48 h compared to PBS-male mice during colitis. By contrast, DSS-mediated colitis was associated with elevated DAI scores at 72 and 96 h (*p* < 0.05) along with early and more severe weight loss in PSTi8-treated females compared to in PSTi8-treated male mice and PBS-treated female and male groups. A non-significant difference was observed in the sum of DAI and macro-score in PSTi8-treated females compared to in their PBS-treated female counterparts and PSTi8-treated male mice (Figure 1C,D). Interestingly, PSTi8-treated male mice had significantly higher weight loss occurring 24 h earlier than PBS-treated male mice in colitic conditions.

### 2.2. PST Inhibition Correlated with Reduced Levels of Several Colonic Mucosal Cytokines in Female Mice at a Steady State

**Figure 2:**
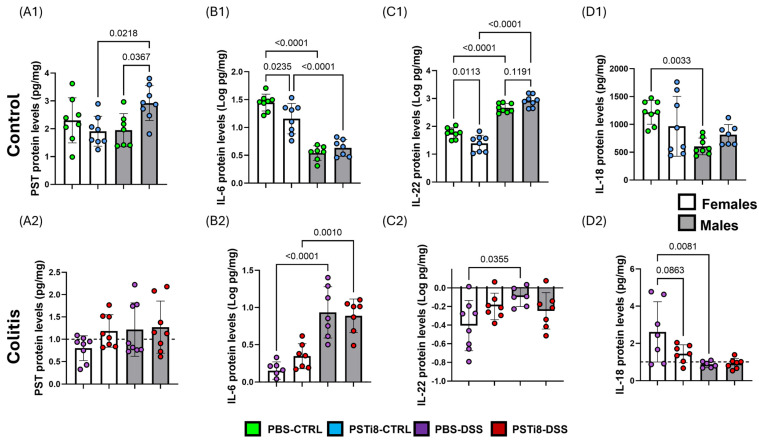
PSTi8 treatment altered the profile of colonic mucosal cytokines in a sex-dependent manner at a steady state and colitic conditions. Male and female C57BL/6 (WT) mice (n = 6–8) were administered with PSTi8 (2.5 mg/kg/day) or PBS by intra-rectal route one day before treatment with 5% DSS for five days to induce colitis. Colon tissues were collected and homogenized to extract proteins. Protein levels of PST (**A1**,**A2**) and mucosal regulatory inflammatory cytokines, interleukin (IL)-6 (**B1**,**B2**), IL-18 (**C1**,**C2**), and IL-22 (**D1**,**D2**), were evaluated by ELISA. Cytokine concentrations in colitic conditions were normalized to control conditions. The dashed line in each figure represents a ratio of 1, indicating no difference between DSS-induced colitis and control conditions. Data were expressed as the mean ± standard deviation. Statistical significance was determined using ordinary one-way ANOVA and Tukey’s multiple comparisons test. Statistical analysis to determine outliers was performed using robust regression and outlier methods.

Given the critical roles played by cytokines in regulating the colonic mucosal barrier integrity and function in homeostatic and pathological conditions, we characterized the impact of PSTi8 treatment on levels of PST, interleukin (IL)-6, interleukin (IL)-18, and interleukin (IL)-22 (Figure 2(A1–D2) and Figure S1). Except for PST, these cytokines are known to modulate mucosal integrity and function in UC in male and female mice at a steady state and during colitis. The levels of IL-6 and IL-22 in colonic tissues from PSTi8-treated females were significantly lower than those measures in tissue from PBS-treated female mice at a steady state (*p* < 0.01). PSTi8 treatment did not alter IL-6 and IL-22 levels in male mice at a steady state. However, the levels of IL-6 and IL-22 were significantly lower and higher, respectively, in PSTi8-treated or PBS-treated males when compared to in PSTi8-treated or PBS-treated female mice at a steady state (*p* < 0.05) (Figure 2(B1,C1)). No significant differences in IL-18 and PST levels were observed between PSTi8-treated and PBS-treated female groups (Figure 2(A1,D1)). However, PSTi8-treated males had significantly higher levels of PST at a steady state than PBS-treated male groups (Figure 2(A1)). Interestingly, PBS-treated male mice had markedly lower IL-18 levels than PBS-treated female groups at a steady state (Figure 2(D1)). The levels of IL-6 and IL-18 did not differ markedly in colonic tissues between PSTi8-treated and PBS-treated male groups in homeostatic conditions.

Colitis in PBS-treated female mice was associated with no significant difference in IL-6 and significantly higher IL-18 levels, whereas PST and IL-22 expression were markedly reduced (*p* < 0.05) compared to in the non-colitic PBS-treated female groups (Figure S2A,B,E,F). However, no significant differences in PST, IL-6, IL-22, and IL-18 levels were observed between PSTi8-treated and PBS-treated female groups in colitic conditions (Figure 2(A2–D2)). In PBS-treated males, colitis resulted in markedly elevated IL-6 and no significant changes in PST, IL-22, and IL-18 levels compared to in the corresponding non-colitic PBS-treated mice (Figure S1E–H). Moreover, the levels of all four cytokines were not altered significantly between PSTi8-treated and PBS-treated male mice in colitic conditions.

### 2.3. PST Inhibition Is Associated with Changes in the Expression of Several Colonic Mucosal Repair Markers Between Sexes That Differ at Steady State and Colitic Conditions

**Figure 3:**
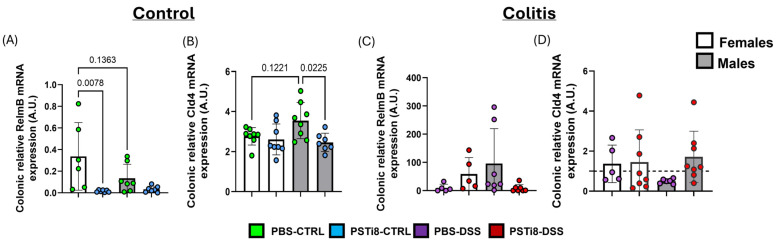
PST inhibition modulated the expression of markers associated with mucosal integrity at a steady state, but not in colitic conditions. (**A**–**D**) Colonic tissue collected from males and female C57BL/6 (WT) (n = 4–8) treated intrarectally with 2.5 mg/kg/day PSTi8 or PBS daily for 6 days concomitant with DSS or water for 5 days were used (control group, CTRL). (**B**–**D**) mRNA expression levels of markers linked to microbiota mucosal colonization, Resistin-like protein β (realmβ), and tight junction, claudin 4 (Cld4) were measured by qRT-qPCR. Data obtained from DSS-treated mice were normalized to control conditions. The dashed line in each figure represents a ratio of 1, indicating no difference between DSS-induced colitis and control conditions. Results represented the means ± standard deviation. One-way ANOVA followed by Tukey’s post hoc was performed to compare group averages.. Statistical analysis using robust regression and outliers was performed to identify outliers.

To investigate whether PSTi8 treatment altered the colonic mucosal barrier function, we characterized the mRNA expression of TJ protein claudin 4 *(Cld4*) as well as cytokine Resistin-like protein β (*Relmβ*), used here as an indicator of colonic epithelial cells’ response to colonic mucosa colonization by microbiota [34]. PSTi8 treatment altered the expression of markers associated with mucosal integrity differentially in male and female mice at a steady state and colitic conditions (Figure 3 and Figure S2). PSTi8-treated female groups had significantly lower *Relmβ* levels (*p* < 0.008) and no significant changes in *Cld4* expression when compared to PBS-treated female groups at a steady state (Figure 3A,B). By contrast, PSTi8-treated male mice exhibited reduced *Cld4* mRNA expression (*p* < 0.02) and a non-significant difference in *Relmβ* transcript level at a steady state. Although only a minor difference in *Relmβ* transcript levels was observed in PBS-treated female mice between colitic and steady-state conditions, PSTi8-treated female mice presented with significantly elevated *Relmβ* transcript levels in colitic as compared to under steady-state conditions (*p* < 0.02) (Figure S2A). However, no significant difference in *Relmβ* and *Cld4* mRNA expression levels was observed between PSTi8- and PBS-treated female groups in colitic conditions (Figure 3C,D). By contrast, PBS-treated male mice had significantly elevated *Relmβ* and lower *Cld4* mRNA expressions in colitis as compared to in steady-state conditions (*p* < 0.04) (Figure S2C,D). However, PSTi8 treatment altered *Relmβ* and *Cld4* mRNA expressions. Hence, PSTi8-treated male mice had a trend toward reduced *Relmβ* and a significant increase in *Cld4* mRNA expression (*p* = 0.04) in colitic compared to in steady-state conditions (Figure S2C,D). Interestingly, despite showing a trend toward reduced *Relmβ* transcript levels, *Relmβ* and *Cld4* mRNA expressions did not differ significantly between PSTi8-treated and PBS-treated male mice in colitic conditions (Figure 3C,D).

### 2.4. PST Inhibition Is Associated with No Significant Changes in the Colonic Mucosal Antimicrobial Activities in Male and Female Mice at Steady State and Colitic Conditions

As presented above, we demonstrated that PSTi8 treatment correlated with significant regulation of IL-22 levels and *Relmβ* expression at a steady state and to a lesser extent in colitic conditions. Therefore, we investigated whether PSTi8 could regulate mucosal antimicrobial activities at a steady state and colitic conditions. We characterized antimicrobial peptides Ly6/Plaur domain containing 8 (*Lypd8*), WAP four-disulfide core domain protein (*Wfdc2*), cathelin-related antimicrobial peptide (*Cramp*), regenerating islet-derived protein 3 β *(RegIIIβ*), and *RegIIIγ* mRNA expressions. PSTi8 treatment was not associated with altered mRNA expression of all tested antimicrobial peptides in both male and female mice at a steady state (Figure S3). *Cramp* and *RegIIIβ* transcript levels were significantly increased in colonic tissues from PBS-treated female mice in colitic compared to in non-colitic conditions (Figure S3(C1,D1)). Levels of gene transcripts of *Cramp* and, to a lesser extent, *RegIIIβ*, were elevated in tissue from PSTi8-treated female mice in colitic compared to in non-colitic conditions. No significative differences in the transcript level of all tested antimicrobial peptides were observed between PSTi8-treated female mice and their PBS-treated female counterpart in colitic conditions (Figure S3(F2–J2)). By contrast, PBS-treated males had significantly higher level of Lypd8 transcripts in colitic compared to in non-colitic conditions (*p* < 0.0001) (Figure S3(F1)). However, although not significative*, Lypd8* and *Wfdc2* transcript levels were lower in PSTi8-treated male mice than in PBS-treated male mice in colitic conditions. Higher *Cramp* mRNA expression and a trend toward elevated *RegIIIβ* and *RegIIIγ* were observed in colitic compared to in non-colitic PSTi8-treated male mice (Figure S3(H1–J1)). No significant difference in *Cramp*, *RegIIIβ*, and *RegIIIγ* expression between colitic PSTi8- and PBS-treated male mice was observed (Figure S3(H2–J2)).

### 2.5. PST Inhibition Is Associated with Changes in the Expression of Colonic Mucosal Repair Markers Between Sexes at Steady State and During Colitis

**Figure 4:**
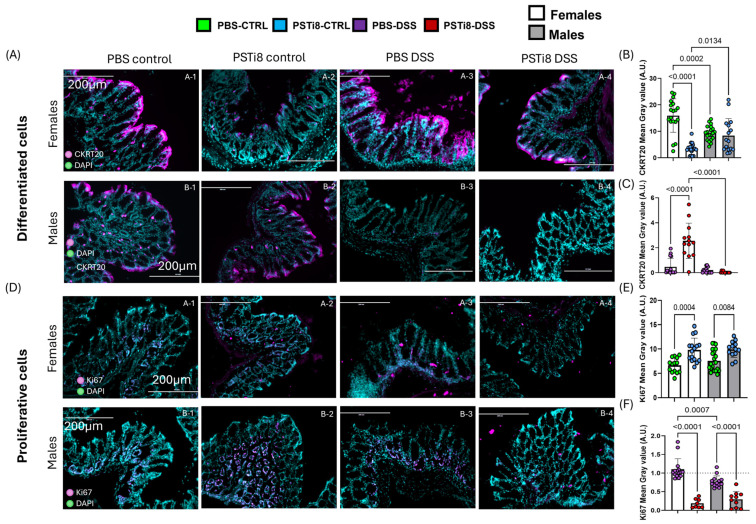
PST inhibition correlated with altered expression of colonic mucosal repair markers between sexes at a steady state and colitic conditions. (**A**–**F**) Colonic tissue collected from males and female C57BL/6 (WT) (n = 3) treated intrarectally with 2.5 mg/kg/day PSTi8 or PBS daily for 6 days concomitant with DSS or water for 5 days were used (control group, CTRL). Protein levels of proliferation (KI67) (**A**–**C**) and differentiation (cytokeratin 20, CKRT20) markers (**D**–**F**) were assessed by immunofluorescence (IF). Scale bar: 200 µm. Data from DSS-treated mice were normalized to control conditions. The dashed line in each figure represents a ratio of 1, indicating no difference between DSS-induced colitis and control conditions. Results represented the means ± standard deviation. One-way ANOVA followed by Tukey’s post hoc was performed between groups. Statistical analysis using robust regression and outliers was performed to identify outliers.

Immunofluorescence analysis to assess the colonic mucosal repair revealed a significantly lower level of cytokeratin 20 (CKRT20), a marker of differentiation in the colon (24), along with increased levels of the proliferative marker KI67 in PSTi8-treated female mice in comparison to in the PBS-treated female mice at a steady state (*p* < 0.0001 and *p* = 0.0004 respectively) (Figure 4A,B,D,E). KI67 levels were also significantly higher in colon tissue from PSTi8-treated male mice than PBS-treated male mice at a steady state (*p* = 0.0008) (Figure 4D,E). DSS-mediated colitis was associated with a marked reduction in CKRT20 levels (*p* < 0.03 to <0.0001) and nonsignificant differences in KI67 levels in colitic PBS-treated female and male mice in comparison to in their non-colitic PBS-treated counterparts (Figure 4A,C,D,F). Similarly, expression levels of CKRT20 and KI67 were significantly lower in colitic PSTi8-treated compared to in the non-colitic PSTi8-treated females and male mice (*p* < 0.0001). Interestingly, KI67 levels were more significantly reduced in colitic PSTi8-treated female and male mice compared to in colitic PBS-treated female and male mice (*p* < 0.0001) (Figure 4F).

### 2.6. PST Inhibition Is Associated with Levels of Colonic Differentiated Epithelial Cells That Differ in a Sex-Dependent Manner at Steady State and Colitic Conditions

**Figure 5:**
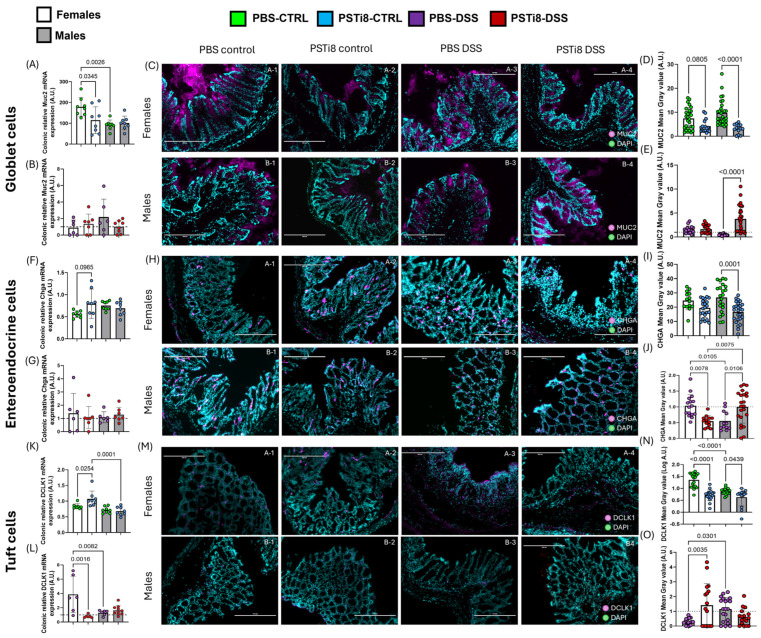
PSTi8 treatment was associated with changes in the expression of several terminally differentiated colonic epithelial cell markers between sexes at a steady state and colitic conditions. Expression of markers specific to differentiated colonic epithelial cells was evaluated. (**A**–**O**) Colonic tissues collected from males and female C57BL/6 (WT) (n = 5–8) treated intrarectally with 2.5 mg/kg/day PSTi8 or PBS daily for 6 days concomitant with DSS or water for 5 days were used (control group, CTRL). mRNA and protein levels of markers specific to goblet cells (**A**–**E**), mucin (MUC2) 2, enteroendocrine cells (**F**–**J**), chromogranin A (CHGA), and Tuft cells (**K**–**O**), doublecortin-like kinase (DCLK) 1, were characterized by RT-qPCR and immunofluorescence (IF) (n = 3). Scale bar: 200 µm. Data from DSS-treated mice were normalized to data from control mice. The dashed line in each figure represents a ratio of 1, indicating no difference between DSS-induced colitis and control conditions. The histogram represents the means ± standard deviation. One-way ANOVA followed by Tukey’s post hoc was performed between groups. Statistical analysis using robust regression and outliers was performed to identify outliers.

Our finding suggests that PST inhibition may regulate the profile of terminally differentiated epithelial cells at a steady state and during colitis. We characterized the levels of mucin 2 (MUC2), CHGA, and double cortin-like kinase 1 (DCLK1) markers for goblet, enteroendocrine, and tuft cells, respectively, in colonic tissues from PSTi8-treated male and female mice in homeostatic and colitic conditions. qRT-PCR and immunofluorescence analysis revealed significantly lower *Muc2* RNA expression in PSTi8-treated females and reduced MUC2 protein levels in PSTi8-treated male mice compared to in their PBS-treated counterpart at steady state (*p* < 0.05; *p* < 0.0001) (Figure 5A,C,D). Despite showing a trend toward lower MUC2 protein levels, no significative differences in MUC2 expression were observed at protein levels in PSTI8-treated females and at mRNA levels in PSTI8-treated male mice. No marked differences in the transcript and protein expression of CHGA were observed in PSTi8-treated female mice compared to in PBS-treated female mice. By contrast, CHGA protein levels were significantly lower in PSTi8-treated male mice in comparison to in PBS-treated male mice at a steady state (Figure 5F,H,I). We also find that DCLK1 levels were markedly lower in PSTi8-treated female and male mice than in their PBS-treated counterparts at the protein level in both sexes and at an mRNA level in males (Figure 5K,M,N).

Colitis was associated with no significant changes in *Muc2*, *Chga*, and the colonocyte marker, carbonic anhydrase *(CA)2*, mRNA levels, and increased Dclk1 expression (*p* = 0.02) in PSTi8-treated female mice compared to in PSTi8-treated female mice in non-colitic conditions (Figure S4(A1–D1)). However, differentiated epithelial cell marker levels were substantially altered at protein levels in colitis (Figure S4(A2–C2)). Thus, results showed increased MUC2 along with decreased DCLK1 levels in PBS-treated females in colitic compared to in non-colitic conditions (*p* < 0.05) (Figure S4(A2,C2)). On the other hand, PSTi8-treated female mice displayed significantly reduced CHGA and elevated DCLK1 levels in colitis in comparison to in non-colitic conditions (*p* < 0.01) (Figure S4(B2,C2)). When normalized to their control conditions at a steady state, PSTi8-treated female mice presented with no significant difference in the gene expression of most differentiated epithelial cells except for the marked reduction in *Dclk1* mRNA expression compared to PBS-treated females and PSTi8-treated male mice (Figure 5B,G,L). However, protein levels of CHGA declined, and those of Dclk1 increased in PSTi8-treated female mice compared to in PBS-treated female mice in colitis (Figure 5E,J,O).

PBS-treated male mice had increased *Dclk1* and *Ca2* mRNA expression (*p* < 0.05) and no significant changes in *Muc2* and *Chga* gene expression in colitic compared to in non-colitic conditions (Figure S4(E1,H1)). However, MUC2 and CHGA protein levels were significantly diminished in PBS-treated male mice in colitis compared to in steady-state conditions (Figure S4(E2,F2)) (*p* = 0.04; *p* = 0.0009). On the other hand, PSTi8-treated male mice did not display significant changes in the gene expression of all four markers compared to their PBS-treated counterparts in colitic conditions. However, at the protein level, we found that PSTi8-treated male mice had significantly higher MUC2 levels in colitic compared to in non-colitic conditions (*p* = 0.0001) (Figure S2(E2)) along with increases in MUC2 and CHGA levels compared to colitic PBS-treated male mice (*p* < 0.05) (Figure 5E,J).

### 2.7. PST Inhibition Is Associated with Changes in Several Stem Cell Populations in a Sex-Dependent Manner at Steady State and During Colitis

**Figure 6:**
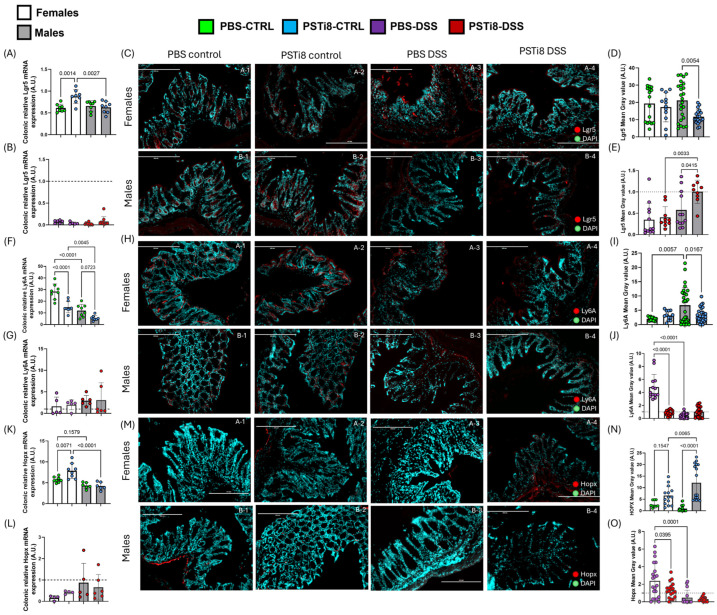
PST inhibition correlated with an altered profile of several colonic stem cells in a sex-dependent manner at a steady state and colitic conditions. The expression levels of markers associated with colonic epithelial stem cells were evaluated. Male and female C57BL/6 (WT) (n = 4–8 mice per group) were utilized for this study. Mice were treated with intrarectal injection with 2.5 mg/kg/day PSTi8 or PBS daily for 6 days. One day after starting PSTi8 treatment, mice received, for five consecutive days, 5% dextran sulfate sodium (DSS) added in the drinking water to induce colitis or with water for the control group (CTRL). Colon tissue extracted at day five post-DSS treatment was analyzed using RT-qPCR and immunofluorescence (n = 3). Scale bar: 200 µm. Biomarkers specific to crypt base columnar (LGR5) (**A**–**E**), paligenosis (fetal-like) (LY6A) (**F**–**J**), and damage-associated regenerative (HOPX) colonic stem cells (**K**–**O**) were evaluated. Data from DSS-treated mice were normalized to control conditions. The dashed line in each figure represents a ratio of 1, indicating no difference between DSS-induced colitis and control conditions. The histogram represents the mean ± standard deviation. One-way ANOVA followed by Tukey’s post hoc was performed between groups. Statistical analysis using robust regression and outliers was performed to identify outliers.

We explore whether the altered colonic mucosa proliferative activity observed at a steady state and in colitis, as demonstrated in Figure 4, resulted from modifications in the profile of the stem cell population. Thus, as shown in Figure 6, PSTi8-treated female mice had significantly elevated *Lgr5* and *Hopx* expression levels and lower *Ly6a* mRNA expression than the PBS-treated female groups at a steady state (*p* < 0.01) (Figure 6A,C,D,F,H,I,K,M,N). However, no significative changes in the levels of all three stem cell markers were observed at the protein level at a steady state. Conversely, although no significant changes in the expression levels of all three markers were observed at the transcript level, protein levels of LGR5 and LY6A were lower in PSTi8-treated male mice compared to in PBS-treated male mice (*p* < 0.05). By contrast, Hopx levels were higher in PSTi8-treated male mice compared to PBS-treated male mice at a steady state (*p* < 0.05).

PBS-treated female mice displayed a significant downregulation of LGR5 expression (*p* < 0.001) and increased LY6A expression in colitic compared to in no-colitic conditions (*p* < 0.001) (Figure S5(A1–B2)). No changes in HOPX expression were observed at the mRNA and protein levels in colitic compared to in non-colitic conditions (Figure S5(C1,C2)). Similarly, LGR5 expression at the mRNA and protein levels diminished in colitic PSTi8-treated female mice compared to in their non-colitic PSTi8-treated female counterparts (*p* < 0.05; Figure S5). However, no significant differences in the level of LY6A and HOPX were observed between colitic and non-colitic PSTi8-treated female mice. *Lgr5*, *Ly6a*, and *Hopx* gene expression did not differ between PSTi8-treated and PBS-treated female mice during colitis (Figure 6B,G,L). However, PSTi8-treated female mice had significantly reduced LY6A and HOPX protein levels compared to PBS-treated female mice during colitis (*p* < 0.05) (Figure 6H,M,J,O).

During colitis, *Lgr5a* and *Hopx* mRNA expression levels were markedly reduced, while Ly6a expression remains unaltered in both PSTi8-treated and PBS-treated male mice in comparison to in their non-colitic counterparts (*p* < 0.0001) (Figure S5(D1,F1)). The mRNA results were, in part, validated at the protein level. Thus, LGR5 protein levels were reduced in colitic PBS-treated male mice, whereas HOPX levels declined in colitic PSTi8-treated male mice compared to in their PSTi8-treated non-colitic counterparts (Figure S5(D2–E2)). However, unlike mRNA results, LY6A protein levels were downregulated in PBS-treated male mice (<0.0001). In contrast, no significant differences in all three stem cell markers were observed in PSTi8-treated male mice between colitic and steady-state conditions (Figure S5(E2,F2)). The transcript levels of all three stem cell markers did not differ between colitic PBS-treated and PSTi8-treated male mice. However, PSTi8-treated male mice preserved *Lgr5* protein levels compared to PBS-treated mice (Figure 6E).

### 2.8. PST Inhibition Correlates with Altered Expression of Colonic Mucosal Self-Renewal and Differentiation Markers Between Sexes at Steady-State and Colitic Conditions

**Figure 7:**
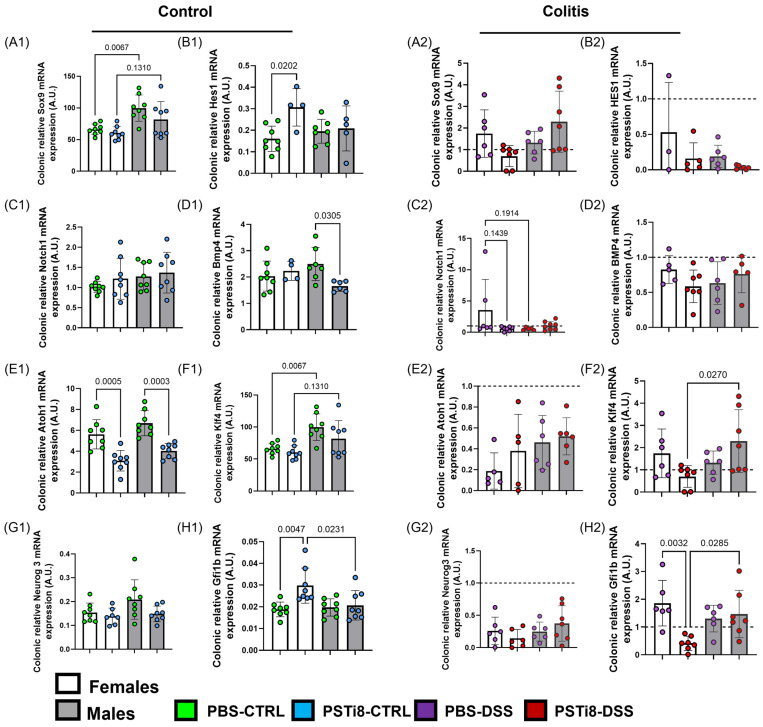
PST inhibition was associated with a change in the expression levels of several colonic epithelial cell lineage commitment markers between sexes at a steady state and colitic conditions. Males and females C57BL/6 (WT) (n = 4–8) were administered with PSTi8 (2.5 mg/kg/day) or PBS by intra-rectal route one day before treatment with 5% DSS to induce colitis. mRNA expression levels of markers specific for transcription factors mediating stem cell renewal *Sox9*, *Hes1* (**A1**–**B2**), absorptive progenitors (*Notch1*) (**C1**,**C2**), differentiation (**D1**,**D2**), secretory progenitors (Atoh1) along with differentiation of goblet cells (*Klf4*) (**E1**–**F2**), enteroendocrine *Neurog* (**G1**,**G2**), and Tuft cells, *Gfi1b* (**H1**,**H2**) were measured by RT-qPCR. Data from DSS-treated mice were normalized to control conditions. The dashed line in each figure represents a ratio of 1, indicating no difference between DSS-induced colitis and control conditions. The histogram data represent the mean ± standard deviation. One-way and two-way ANOVA followed by a multiple comparison test were assessed. One-way ANOVA followed by Tukey’s post hoc was performed between groups. The statistical analysis using robust regression and outliers was performed to identify outliers.

To investigate the mechanism underlying PSTi8 effect on colonic epithelial cells lineage commitments, transcription factors including NOTCH1, bone morphogenetic protein (BMP), hairy and enhancer of split-1 (Hes1), atonal homolog 1 (Atoh1), Sry-related HMG box 9 (SOX9), Krüppel-like factor 4 (KLF4), Neurogenin3 (NEUROG3), and Growth factor independence 1b (GFI1b) were characterized. The results showed that *Hes1* and *Gfi1b* mRNA expression levels were higher while *Atoh1* transcript expression was reduced in colon tissues of PSTi8-treated female mice compared to PBS-treated female mice at a steady state (*p* = 0.02, 0005, and 0.005) (Figure 7(B1,E1,H1)). By contrast, in males, colonic tissues from PSTi8-treated mice had significantly lower *Bmp4* and *Atoh1* expression compared with those from PBS-treated mice (*p* = 0.03 and 0.0003) (Figure 7(D1,E1)). PBS- and PSTi8-treated male mice expressed markedly higher levels of *Sox9* and *Klf4* transcripts than their PBS- and PSTi8-treated female counterparts at a steady state (Figure 7(A1,F1)).

We further characterized the effects of PSTi8 on colonic epithelial cells lineage commitment in colitic conditions (Figure 7 and Figure S6). *Hes1*, *Bmp4*, and *neurog3* mRNA expression levels were significantly downregulated in colonic tissues from both PSTi8- and PBS-treated female mice in colitis compared to in non-colitic conditions (Figure S6). *Atoh1* expression was elevated, whereas *Klf4* and *Gfi1b* mRNA expressions were lower in colonic tissues from PBS-treated female mice in colitic compared to in non-colitic conditions. By contrast, significantly lower *Gfi1b* mRNA expression was observed in tissue from PSTi8-treated female mice in colitic compared to in non-colitic conditions (Figure S6(H1)). No significant differences were observed between PSTi8-treated and PBS-treated female mice in colitic conditions (Figure 7(A2–H2)). Similar to females, *Hes1*, *Atoh1*, and *Neurog3* transcript levels were significantly reduced in colon tissue from both PSTi8- and PBS-treated male mice in colitic compared to in non-colitic conditions (Figure S6(B2,E2,G2)). Moreover, no significant differences were observed in *Sox9*, *Notch1*, *Klf4*, and *Gfi1b* mRNA expression levels in tissues from colitic PBS-treated male mice; *Sox9* and *Klf4* transcript levels were significantly upregulated in colon tissues from PSTi8-treated male mice in colitic compared to in non-colitic conditions (*p* ≤ 0.04 and 0.02) (Figure 7(A2–H2)). No marked differences in the expression of all transcription factors were observed between PSTi8- and PBS-treated male mice in colitis, whereas *Klf4* and *Gfi1b* expression levels were significantly higher in PSTi8-treated male than in their female counterparts in colitis (Figure 7(F2,H2)).

### 2.9. PSTi8 Treatment Correlates with Changes in the Expression of Several UPR Signaling Markers in Female Mice at Steady State and Colitic Conditions

**Figure 8:**
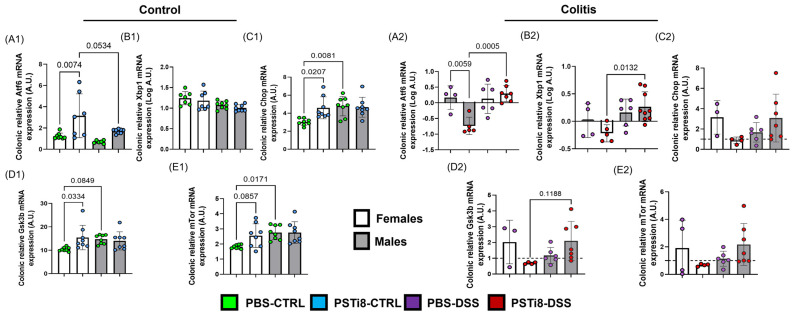
PST inhibition was associated with changes in the expression of several endoplasmic reticulum (ER) stress, mediators of apoptosis, and proliferation markers in a sex-dependent manner at a steady state. Colon tissues from males and females C57BL/6 (WT) (n = 3–8) administered with PSTi8 (2.5 mg/kg/day) or PBS by intra-rectal route one day before treatment with 5% DSS to induce colitis will be used. Biomarkers specific to ER stress (*Atf6*, *Xbp1*) (**A1**–**B2**), apoptosis (*Chop*) (**C1**,**C2**), and inhibition of cell growth *(mTor*, *Gsk3b*) (**D1**–**E2**) were assessed from colon samples by RT-qPCR. Data from DSS-treated mice were normalized to control conditions. The dashed line in each figure represents a ratio of 1, indicating no difference between DSS-induced colitis and control conditions. The histogram data represent the mean ± standard deviation. One-way ANOVA followed by a multiple comparison test was performed. One-way ANOVA followed by Tukey’s post hoc was performed between groups. The statistical analysis using robust regression and outliers was performed to identify outliers.

Finally, we investigated whether PSTi8 regulated GRP78 downstream signaling and assessed activating transcription factor (ATF)6, X-box binding protein (XBP)1, C/EBP Homologous Protein (CHOP), glycogen synthase kinase (GSK)3β, and mammalian target of rapamycin (MTOR) expression in colonic tissue from PSTi8- and PBS-treated male and female mice at a steady state and colitic conditions. qRT-PCR analysis revealed that *Atf6*, *Chop*, *Gsk3β*, and *mTor* expression levels were significantly increased in PSTi8-treated female mice than PBS-treated female mice to reach the higher levels observed in all-male groups at a steady state (Figure 8(A1,C1–E1)). Although not significative, Atf6 elevated expression in PSTi8-treated female mice was superior to levels observed in PSTi8-treated male mice, while *Xbp1* expression did not differ between both groups at a steady state (Figure 8(A1,B1)). No significant differences in *Atf6*, *Xbp1*, *Chop*, *Gsk3β*, and *mTor* were observed in all-male groups at a steady state.

When evaluated in colitic conditions, PBS-treated females had significantly more Chop and a trend toward elevated Gsk3β expression when compared to their female counterparts in non-colitic conditions (*p* = 0.0004 and 0.0532) (Figure S7(B1,C1)). By contrast, no significant changes in *Chop* and *Gsk3β* were observed in colitic compared to in non-colitic PBS-treated male mice (Figure S7(B2,C2)). No significant differences in the expression of all four transcription factors were measured between PST8-treated female mice in colitic and non-colitic conditions, whereas *Atf6*, *Xbp1*, *Chop*, *Gsk3β*, and *mTor* expression levels were all significantly upregulated in PSTi8-treated male mice in colitic compared to in non-colitic conditions (for all transcription factors: *p* < 0.05). PSTi8 treatment correlated with altered mRNA expression levels of some of GRP78 downstream signaling proteins that differed in a sex-dependent manner, with significantly lower Atf6 and a trend toward reduced *Xbp1*, *Chop*, and *Gsk3-β* in PSTi8-treated females compared to in PBS-treated females and more marked compared to PSTi8-treated male mice, and no difference was observed across colitic male groups (*p* = 0.01 and 0.0005) (Figure 8(A2–E2)).

## 3. Discussion

The colonic mucosa produces over 50 hormones and biologically active peptides, many of which are secreted by enteroendocrine cells [35]. As part of the endocrine system, these peptide hormones can mediate diverse and contrasting physiological actions systematically by regulating the immune cells and the enteric nervous system or locally by regulating secretion, motility, insulin release, and nutrient uptake in the GI tract [36]. Although a growing number of studies described a role for enteroendocrine peptide hormones in the context of metabolic physiological and pathological conditions such as obesity and diabetes, the effects of enteroendocrine secreted peptide hormones on colonic mucosal and epithelial cells integrity, profile, and functions in inflammatory disorders such as UC are yet to be fully understood. The current study demonstrated that the colonic mucosal inhibition of PST, an enteroendocrine peptide highly expressed in colonic tissues of patients with active UC and in a mice model of colitis, with PSTi8 treatment altered the epithelium repair process homeostasis and influenced moderately susceptibility to DSS-mediated colitis in a sex-dependent manner [27,37].

Few studies have recently reported that PST regulates intestinal mucosal permeability at a steady state, promotes inflammatory response and exacerbates disease severity in male mice with DSS-mediated colitis [27,29]. Thus, PST treatment in male mice promoted the early onset of colitis and increased the colon mRNA expression of proinflammatory cytokines IL-8 and IL-18 by reducing the alternative activated macrophage activity and expression levels of TJ proteins CLD1, zonula occludens-1, occludin, and E-cadherin-1 during DSS-mediated colitis [27]. Similarly, male mice treated with PSTi8 showed a trend toward delays in colitis onset. However, these results differed in female mice as PSTi8 treatment with moderately worsened colitis progression, suggesting a role for sex-based differences in the response of colonic mucosa to PST.

The repair process of colonic mucosal is orchestrated through a series of events involving a complex interplay between several key players that regulate mucosal barrier integrity, such as cytokines, TJ proteins, and antimicrobial peptides [16,38,39,40,41,42]. During colitis, the release of several proinflammatory mediators, such as IL-6, IL-18, IL-22, and RELMβ, has been shown to enhance the intestinal epithelial cells proliferation, induce the release of antimicrobial peptides and anti-inflammatory mediators and promote the mucosa wound healing [20,40,41,43,44,45,46]. Interestingly, PSTi8-treated groups displayed no significant differences in the production of proinflammatory mediators, PST, IL-6, IL-22, IL-18, and mRNA expression of TJ *Cld4*, goblet-derived bacterially induced *Relmβ*, and antimicrobial peptides in colitic conditions. However, in homeostatic conditions, PST inhibition with PSTi8 correlated with changes in several markers associated with mucosal integrity in a sex-dependent manner. Thus, PST inhibition in females was associated with lower levels of IL-6 and IL-22, along with reduced *Relmβ* mRNA expression, while promoting the production of PST and reducing *Cld4* mRNA expression in males in homeostatic conditions. Moreover, PSTi8 treatment did not alter the expression of antimicrobial peptides at a steady state, indicating a minimal influence of PST on colonic mucosa antimicrobial activities. These observations suggest a potential role of PST as a regulator of mucosa barrier integrity, whose effect is more marked in females than in males.

Cytokines are critical regulators of colonic epithelial barrier integrity as they can induce or restrict epithelial cell death, proliferation, and differentiation and consequently affect the colon’s capacity for wound healing [47]. The epithelial cell restitution, proliferation, and differentiation are primordial mechanisms in the mucosa wound repair process critical to preserving the epithelial barrier function in colon homeostasis [48]. The restitution process requires the migration of surrounding colonic epithelial cells to reseal the injury site and initiate healing without proliferation [48]. The proliferation process increases the pool of colonic epithelial cells necessary to compensate for the cells shed or destroyed during injury. In contrast, differentiation and maturation help to preserve and reestablish the mucosal barrier integrity [38]. Insufficient wound healing during colitis can lead to abscess and fistula, whereas excessive wound healing can cause intestinal fibrosis [49]. Moreover, enhanced proliferation increases the risk for patients to develop colorectal cancer, which is more prevalent in males than females [50,51,52,53]. Therefore, the altered mucosal differentiation observed in females could result in part from the diminished production of proinflammatory cytokines following PSTi8 treatment at a steady state. Along the same line, regulatory anti-inflammatory signaling reduces differentiation and enhances intestinal stem cell renewal [52]. Moreover, we reported that PST decreased the alternatively activated macrophage (AAM) ability to produce anti-inflammatory mediators in colitic conditions. Therefore, PST inhibition may restore AAM and hence the production of anti-inflammatory cytokines [27]. Further studies will be needed to investigate whether PST inhibition through enhanced production of anti-inflammatory cytokines can regulate colonic mucosal cell proliferation and differentiation at a steady state.

We further observed that the inhibitory effect of PSTi8 treatment in females’ colonic mucosal differentiation was associated with substantial tuft cell reduction and a trend toward lower goblet cells at a steady state, indicating that PST likely regulates tuft and goblet cell homeostasis in females’ colon. PSTi8 treatment in males correlated with a marked reduction in goblet, enteroendocrine, and tuft cells, but no significant changes in colonocytes. The lack of changes in the colonocyte population could, therefore, explain the absence of changes in overall mucosal differentiation observed in males at a steady state. Our results showed that in colitis, PSTi8 treatment correlated with differences in the distribution of several differentiated epithelial cells in a sex-dependent manner. Thus, PSTi8 treatment was associated with reduced goblet and enteroendocrine cells but increased tuft cells in colonic epithelium, revealing tuft cells as one of the potential actors in the overall increase in mucosa proliferation during colitis in females (Figure 4C). A recent study correlated the presence of tuft cells with epithelial restitution [54]. It would be of interest to investigate whether PSTi8-mediated increase in tuft cells could be a protective mechanism in response to exacerbated inflammation. By contrast, PSTi8 treatment in males correlated with a conserved number of enteroendocrine and colonocyte cell populations and increased goblet cells while displaying a non-significative reduction of tuft cells during colitis. The distinct effect of PSTi8 treatment on the distribution of each specific differentiated colonic cell could explain the lack of changes in the mucosa differentiation observed in males following treatment during colitis. These results suggest that PST likely regulates the mucosa differentiation capacity by altering the profile of differentiated epithelial cell populations at a steady state and during DSS-mediated acute inflammation in a sex-dependent manner.

With a relatively short lifespan, the colonic epithelium requires constant self-renewal to replenish depleted differentiated cells and preserve the colonic mucosal homeostasis [1]. The self-renewal of colonic epithelial cells is driven primarily by CBCs and DARSCs stem cells LGR5^+^, LY6A^+^, and HOPX^+^ [1,55]. Although dispensable, Lgr5^+^ cells can give rise to all differentiated epithelial cells and, owing to their fast-cycling nature, promote rapid and daily epithelium homeostatic regeneration [56]. However, these stem cells are exquisitely sensitive to cell death during injuries such as radiation, ischemia, or colitis [57]. The DARSCs LY6A^+^ and HOPX^+^ cells arise in response to severe colonic mucosa injury and LGR5^+^ cell depletion to restore the epithelium integrity [1,55,57]. Moreover, all three stem cells can give rise to all terminally differentiated epithelial cells in the colon [1]. Here, we found that PSTi8 treatment was associated with changes in the colonic epithelial stem cell populations differently at a steady state and colitic conditions between sexes. Thus, despite showing a trend toward increased HOPX^+^ cells, PSTi8 treatment did not significantly modulate the levels of all three stem cell populations in females at a steady state. By contrast, this treatment in males correlated with elevated HOPX^+^ cells, in line with the higher overall mucosa proliferation observed in Figure 4E, but lower LGR5^+^ and LY6A^+^ cells at a steady state. These results suggest that PST may regulate HOPX^+^ cell homeostasis in males and females. By contrast, in females, the lack of stem cell modulation associated with the increase in mucosal proliferative activity suggests that PST could regulate other DARSC subtypes, such as CLU^+^ cells shown to regenerate depleted LGR5^+^ cells and help restore the mucosa in acute and chronic DSS-induced colitis [1]. PSTi8 treatment in colitic conditions was associated with a marked reduction in LY6A^+^ and HOPX^+^ cells. The heightened depletion of all three stem cells in females might be responsible for the colonic epithelial cells’ reduced proliferative capacity observed in Figure 3 and Figure 4 and likely explains females’ increased susceptibility to colitis. PSTi8 treatment in males preserved the Lgr5^+^ cell population while depleting HOPX^+^ and LY6A^+^ cells in colitis. Further studies will help decipher whether the ability of males treated with PSTi8 to maintain LGR5^+^ cells, which are fast-cycling stem cells, could increase the regenerative capacity of colonic epithelial cells in colitic conditions.

Several transcription markers promote colonic epithelial cells stemness (SOX9) [58], self-renewal and colonocytes (HES1 and NOTCH1) [2,59], differentiation (BMP4) [60], secretory phenotype (ATOH1), as well as goblet cells (Klf4) [61], enteroendocrine cells (NEUROG3) [62], and tuft cell (GFI1b) [63] lineage commitment. We demonstrated that PSTi8 treatment was associated with elevated *Hes1* and *Gfi1b* mRNA expression and reduced *Atoh1* levels in females in homeostatic conditions. Long thought as a marker of normal colon stem cells, HES1 expression has recently been associated with promoting stemness in a subset of the colonic epithelial cells, in addition to being proposed as a regulator of cancer stem cells in the colon [59]. Given that *Hes1* mRNA expression in the current study does not correlate with the stem cell population regulating the colonic mucosal homeostasis, our data indicate that PSTi8 treatment may enhance the proliferation of unspecified stem cell population potentially associated with cancer stem cells in females at a steady state. GFI1b is a transcription factor specific to a subset of the tuft cell population [63]. We showed that PSTi8 treatment correlated with enhanced *Gfi1b* mRNA expression in females at a steady state. However, this increase did not correlate with the expression of DCLK1^+^ cells, suggesting a potential difference between the population of cells detected by the DCLK1 marker and the one measured using *Gfi1b* mRNA expression. PSTi8 treatment was associated with the reduced mRNA expression of *Atoh1*, a master transcription factor that guides stem cells’ commitment toward a secretory profile, in females and males at a steady state [1]. This decrease in *Atoh1* mRNA expression was likely associated with the reduced goblet and enteroendocrine cell levels observed in females and males in homeostatic conditions. These results highlight a potential role of PST as a regulator of transcription factors that govern the colonic epithelial cell stemness, self-renewal, and differentiation, mediated in a sex-dependent manner.

PST and PSTi8 mechanisms of action occur through GRP78, a molecular chaperon that assists the folding and unfolding of proteins and prevents protein aggregation within the ER [25]. As the master regulator of the UPR signaling induced in response to ER stress, triggered as a result of accumulated misfolded protein in ER, GRP78 controls the availability of ER transmembrane effector proteins inositol requiring enzyme 1 (IRE1) and ATF6 [64]. IRE1 activities generate the potent transactivator of UPR target genes, XBP1, and with ATF6, upregulate GRP78 expression and prevent ER-stress-induced apoptosis mediated by the transcription factor CHOP [64]. Moreover, GRP78 can also regulate cell survival, apoptosis, and systemic inflammation through the enzymes mTOR and GSK3-β [65,66]. ER stress is a hallmark of UC pathophysiology, as it promotes dysregulated autophagy that causes inflammation and impaired intestinal barrier [67]. Our results showed an enhanced UPR response in females treated with PSTi8 at a steady state. However, PSTi8 treatment was not associated with changes in GRP78 signaling during colitis, suggesting the effect of PSTi8 to be mainly mediated in homeostatic conditions in females. Given the critical and complex role of ER stress and UPR signaling in promoting colorectal cancer (CRC), further studies will help understand whether PST regulates CRC etiology in females. PSTi8 treatment did not alter the GRP78 downstream signaling in males at a steady state and during colitis. These results indicate that the effect of PSTi8 on the mucosa in males may not occur through GRP78 receptors and could potentially occur via the IRS, which is also a target receptor for PSTi8 inhibitory action [22,23,24,25,26]. Several studies have shown that estrogen, a female sex hormone, can regulate GRP78 expression in various tissues. Thus, this may explain the dichotomy in the mucosal response to PSTi8 treatment observed between the sexes [28,68,69,70].

This study has some limitations that need further investigation in the future. Firstly, we investigated the expressions of several markers at mRNA levels only. However, transcript levels do not always reflect the translated protein levels, as exemplified by data in Figure 5 and Figure 6. These discrepancies can arise from several pre- and post-transcriptional mechanisms that may influence mRNA stability and impact protein translation and synthesis [71]. Secondly, female mice’s estrus cycle was not synchronized. However, studies have shown that the menstrual cycle and female sex hormones can influence intestinal function and exacerbate disease severity in UC [72]. Third, we only investigated the impact of PSTi8 treatment mediated through the GRP78 signaling pathways. Given the lack of difference in GRP78 downstream signaling in males treated with PSTi8, it will be interesting to investigate the PSTi8-mediated effect on the mucosa through other PST receptors including IRS signaling pathways [28]. Fourth, in the current study, we used an acute model of colitis to characterize the effect of PST inhibition on mucosal integrity. However, this model is associated with excessive inflammation, severe colonic epithelial destruction, and impaired mucosal functions. All these elements could mask the effect of PST on mucosa and impede the capacity to investigate the role of PST in the colonic mucosa. Therefore, evaluation of PST’s role on the mucosa using a chronic DSS model of colitis with low to moderate mucosal destruction may provide more insights into the role of PST in colitis. Finally, gut microbiota plays a significant role in intestinal epithelial homeostasis and UC pathophysiology [73]. Moreover, gut microbiota can also regulate sex hormones, which, in turn, can modulate intestinal integrity [7,74,75,76]. Therefore, further studies will be necessary to confirm the interaction between the colonic epithelium and PST and to investigate the interaction between PSTi8 effects, sex hormones, and microbiota on the colonic mucosa through GRP78 and IR.

In conclusion, the current study suggests that the colonic mucosa inhibition of PST with PSTi8 treatment is associated with changes in the epithelium repair process that differs in a sex-dependent manner at a steady state and could influence, although moderately, the contrasting susceptibility to DSS-mediated colitis observed between female and male mice [37]. Altogether, this study suggested that PST inhibition with PSTi8 could alter the colonic mucosa integrity in females by limiting the differentiation while promoting the proliferation of colonic epithelial cells and increasing UPR signaling pathways in homeostatic conditions. These disturbances of the mucosa in homeostatic conditions could potentially explain the moderate increase in susceptibility to colitis associated with a trend toward worsened disease severity in female mice. By contrast, PSTi8 treatment in male mice resulted in the diminution of epithelial cell proliferation associated with a higher number of HOPX^+^ stem cells and a reduced number of most terminally differentiated epithelial cells goblet, enteroendocrine, and tuft cells at a steady state. However, unlike in females, during colitis, PSTi8 treatment may provide a protective effect on the colonic mucosa against DSS-mediated colitis in male mice as PST inhibition either preserved or promoted the proliferative and differentiation capacity of the colonic epithelium by increasing the number of goblet cells and preserving LGR5^+^ Stem cells and enteroendocrine cells, all of which were overall associated with delay disease onset.

This descriptive study provides new insights regarding the regulatory role of PST in the colonic mucosa integrity and epithelial properties and functions at a steady state and in inflammatory conditions and how its effect differs between sexes. Moreover, we describe the use of PSTi8 as a potential therapeutical tool that limits PST and regulates colonic mucosa repair in UC and could offer a novel therapeutical target for CRC.

## 4. Materials and Methods

### 4.1. Peptides

PSTi8 (PEGKGEQEHSQQKEEEEEMAV-amide) purified by reverse-phase high-performance chromatography to a purity level of <98% was used in this study (Pepmic Co., Suzhou, China).

### 4.2. Mice

All experiments were conducted according to the Canadian guidelines for animal research. For this study, 12-week-old male and female C57BL/6 wild-type mice were obtained from the colonies at the University of Manitoba, Winnipeg, Manitoba. Animals were kept in a 12 h dark/light cycle and fed ad libitum. Forty-eight male and fifty-two female mice were randomly assigned into four groups. The groups included PBS- and PSTi8-treated control male and female groups (PBS-control and PSTi8-control) and PBS- and PSTi8-Dextran sulfate sodium (DSS)-treated groups (PBS-DSS and PSTi8-DSS) mice.

### 4.3. Mice Treatment with Pancreastatin Inhibitor 8 (PSTi8)

Female and male mice were randomly separated into four groups of 12−16 mice. All mice anesthetized with 3% isoflurane and 1L oxygen [25] received daily intra-rectal (i.r.) administration of 2.5 mg/kg/day (n = 28 females and n = 24 male mice), which was shown to have a more prominent inhibitory effect than acute PSTi8 treatment in a mice model of insulin and diabetes [25], or phosphate buffer saline (PBS) (n = 24 females; n = 24 male mice) for six days (modified from previous publications) [25,27].

### 4.4. Induction and Assessment of Experimental Colitis

One day after starting i.r. administration of PSTi8 (n = 16 females; n= 12 males) or PBS (females and males: n = 12), following a well-established protocol, mice were given 5% DSS (molecular weight: 40–50 kDa; ThermoFisher Scientific, Winnipeg, MB, Canada, Catalog #: J14489-22) in their drinking water for 5 days to induce acute colitis [27,77,78]. PSTi8- and PBS-treated control groups (females and males: n = 12) received pasteurized water. Weight loss, stool consistency, and bleeding were monitored daily as previously described and were used to calculate the disease activity index (21). On day five, all anesthetized mice were euthanized by cervical dislocation. Colon macroscopic score was determined using rectal bleeding, rectal prolapse, diarrhea, and colon bleeding (22). The colon collected, clear of feces, segmented into proximal, medial, and distal colons were stored at −80 °C for subsequent assays. The proximal, medial, and distal colons were used for Western blotting, enzyme-linked immunoassay (ELISA), and real-time qPCR (RT-qPCR). Tissue samples were embedded in optimal cutting temperature (OCT) compound (Tissue-Tek, Sakura Finetek USA, Inc., Torrance, CA, USA) and stored at −80 °C for histology and immunofluorescence analysis.

### 4.5. Serum Preparation

Blood samples, allowed to clot at room temperature for 30 min, were centrifuged at 20,000 rpm for 10 min at 4 °C. Supernatants were collected and stored at −20 °C until analyzed.

### 4.6. Procedure for RNA Extraction, Purification, and Quantification

Minced colon samples resuspended in 1mL TRIzol reagent (Invitrogen, ThermoFisher Scientific, Winnipeg, MB, Canada) were mechanically homogenized by sonication, mixed with phenol/chloroform and centrifuged at 12,000 rpm and 4 °C for 20 min. RNA collected in the aqueous phase was diluted with 70% ethanol and purified using an RNA column-based purification kit (PureLink RNA Mini Kit, Invitrogen, ThermoFisher Scientific, Winnipeg, MB, Canada) according to the manufacturer’s instructions. The yield and purity of the extracted RNA eluted in RNAse-free water were assessed using the NanoDrop Spectrophotometer (ThermoFisher Scientific Winnipeg, MB, Canada). The purified RNA samples were reverse transcribed into cDNA using the SuperScript™ IV VILO™ Master Mix (Invitrogen, ThermoFisher Scientific, Winnipeg, MB, Canada) and stored at −80 °C for later analysis.

### 4.7. Epithelial Cells Associated Markers Gene Expression

cDNA samples were analyzed using RT-qPCR on LightCycler 96 instrument (Roche Life Science, Basel, BS, Switzerland) with the Power SYBR™ Green PCR Master Mix (Applied Biosystems™, Life Technologies, Burlington, ON, Canada). The cycle threshold value (Cq) for each gene of interest was normalized to that of the housekeeping gene, TATA-binding protein (TBP), and expressed as the relative ratio of expression. The list of primer sequences is provided in Table 1.

### 4.8. Proteins Extraction

Minced colon samples were homogenized in PBS buffer containing a protease inhibitor cocktail (cOmplete™, Mini Protease Inhibitor Cocktail, Roche, Basel, BS, Switzerland. The homogenate samples were centrifuged at 20,000 rpm for 10 min. Supernatants were collected, and the protein concentrations were measured using the Pierce BCA protein assay according to the manufacturer’s instructions (Bio-Rad Laboratories Inc., Hercules, CA, USA). Samples were stored at −20° C for later analysis.

### 4.9. Cytokines Analysis

Concentrations of interleukin (IL)-6, IL-18 (Duoset, DY206-05 and DY1709, R&D Systems, Minneapolis MN, USA), IL-22 (436304, BioLegend, San Diego, CA, USA), and pancreastatin (MBS267929, MyBiosource, San Diego, CA, USA) were measured by ELISA on extracted proteins from the medial segments of colons according to manufacturers’ instructions.

### 4.10. Immunofluorescence

OCT-embedded frozen tissue sections with a 10 µm thickness were fixed in 4% formalin for 15 min and incubated in a permeabilized solution (0.25% gelatin and 0.2% triton-100X diluted in PBS) for 10 min. The permeabilized tissues were blocked with a blocking solution containing 5% bovine serum albumin (BSA) diluted in permeabilization buffer for 2–4 h at room temperature. Tissues labeled for Muc2 were fixed in Carnoy’s solution (60% absolute ethanol, 30% chloroform, and 10% acetic acid) for 5 min. Moreover, tissues labeled for Lgr5 and Muc2 were not permeabilized. All samples were incubated with a primary antibody diluted in the blocking buffer overnight at 4 °C. Slide sections rinsed in PBS containing 0.1% Triton-100x for 10 min were incubated in appropriate labeled secondary antibodies diluted using the blocking buffer for 1 h and in 4′,6-diamidino-2-phenylindole (DAPI) 1 µg/mL diluted in PBS for 10 min at room temperature in the dark. The stained sections rinsed in washing buffer were incubated in an anti-autofluorescence buffer (10 mM CuSO_4_ and 50 mM NH_4_Cl) and Milli-Q water for 10 min and mounted using the ProLong Gold Antifade Mountant (ThermoFisher Scientific, Winnipeg, MB, Canada). The images acquired using the EVOS Cell Imaging Systems (ThermoFisher Scientific, Winnipeg, MB, Canada) were processed using FIJI 2.16.0 ImageJ software. The list of antibodies used in this study is shown in Table 2.

### 4.11. Statistical Analysis

The statistical analysis was performed with GraphPad Prism Version 9.0.0 software (GraphPad Software, San Diego, CA, USA). Data were expressed as the mean ± standard deviation (S.D.). One-way ANOVA followed by Bonferroni or Tukey-post hoc analysis was performed to calculate the significance between groups. Statistical significance was defined as *p* < 0.05. All data were evaluated for outlier values using robust regression and outlier analysis. Data were log-transformed when the standard deviation was at least three times different between groups with sample sizes smaller than ten or square root-transformed with values equal to 0 [79].

## Figures and Tables

**Table 1:** Narratives and sequences of selected genes.

Target Gene Names		Primer Sequences (5′ to 3′)	PCR Temperature Profile Denature/Anneal/Extend
TATA box protein (*Tbp*)	Forward	ACCGTGAATCTTGGCTGTAAAC	95 °C, 30 s/55 °C, 30 s/72 °C, 45 s
	Reverse	GCAGCAAATCGCTTGGGATTA	95 °C, 30 s/55 °C, 30 s/72 °C, 45 s
**Differentiated epithelial cells**			
Resistin-like molecule β (*RelmB*)	Forward	CCATTTCCTGAGCTTTCTGG	95 °C, 30 s/55 °C, 30 s/72 °C, 45 s
	Reverse	AGCACATCCAGTGACAACCA	95 °C, 30 s/55 °C, 30 s/72 °C, 45 s
Carbonic anhydrases (*Ca2*)	Forward	CAAGCACAACGGACCAGA	95 °C, 30 s/55 °C, 30 s/72 °C, 45 s
	Reverse	ATGAGCAGAGGCTGTAGG	95 °C, 30 s/52 °C, 30 s/72 °C, 45 s
Mucin 2 (*Muc2*)	Forward	GAT GGC ACC TAC CTC GTT GT	95 °C, 30 s/52 °C, 30 s/72 °C, 45 s
	Reverse	GTC CTG GCA CTT GTT GGA AT	95 °C, 30 s/55 °C, 30 s/72 °C, 45 s
Chromogranin A (*Chga*)	Forward	CAGGCTACAAAGCGATCCAG	95 °C, 30 s/55 °C, 30 s/72 °C, 45 s
	Reverse	GCCTCTGTCTTTCCATCTCC	95 °C, 30 s/55 °C, 30 s/72 °C, 45 s
Doublecortin Like Kinase 1 (Dclk1)	Forward	CTGGGTTAATGATGATGGTCTCC	95 °C, 30 s/55 °C, 30 s/72 °C, 45 s
	Reverse	ACAGAAACTCCTGCTGCAGT	95 °C, 30 s/55 °C, 30 s/72 °C, 45 s
**Stem epithelial cells**			
Leucine-rich repeat-containing G-protein coupled receptor 5 *(Lgr5*)	Forward	CTTCCGAATCGTCGATCTTC	95 °C, 30 s/55 °C, 30 s/72 °C, 45 s
	Reverse	AACGATCGCTCTCAGGCTAA	95 °C, 30 s/55 °C, 30 s/72 °C, 45 s
homeodomain-only protein homeobox (*Hopx*)	Forward	TCTCCATCCTTAGTCAGACGC	95 °C, 30 s/59 °C, 30 s/72 °C, 45 s
	Reverse	GGGTGCTTGTTGACCTTGTT	95 °C, 30 s/59 °C, 30 s/72 °C, 45 s
Lymphocyte antigen-6 A (*Ly6a/Sca1*)	Forward	AGGAGGCAGCAGTTATTGTGG	95 °C, 30 s/55 °C, 30 s/72 °C, 45 s
	Reverse	CGTTGACCTTAGTACCCAGGA	95 °C, 30 s/52 °C, 30 s/72 °C, 45 s
**Lineage commitments**			
hairy and enhancer of split 1 (*Hes1*)	Forward	CTTATGAAAGTCAAGTAAAAGGACG	95 °C, 30 s/55 °C, 30 s/72 °C, 45 s
	Reverse	ATAGGCTTTGATGACTTTCTGTG	95 °C, 30 s/55 °C, 30 s/72 °C, 45 s
Atonal homolog 1 (*Atoh1*)	Forward	GACAAATATCCCTGCACCCT	95 °C, 30 s/55 °C, 30 s/72 °C, 45 s
	Reverse	CAGAGGCAGAGATACGACAT	95 °C, 30 s/55 °C, 30 s/72 °C, 45 s
Neurogenic locus notch homolog protein 1 (*Notch 1*)	Forward	CTGAGAGCTCCTGCTTCAAT	95 °C, 30 s/55 °C, 30 s/72 °C, 45 s
	Reverse	AGTACCATAGCTGTCTTGGC	95 °C, 30 s/55 °C, 30 s/72 °C, 45 s
Bone morphogenetic protein 4 (*Bmp4*)	Forward	TTCCTGGTAACCGAATGCTGA	95 °C, 30 s/55 °C, 30 s/72 °C, 45 s
	Reverse	CCTGAATCTCGGCGACTTTTT	95 °C, 30 s/55 °C, 30 s/72 °C, 45 s
Growth Factor Independent 1B (*Gfi1b*)	Forward	ATGCCACGGTCCTTTCTAGTG	95 °C, 30 s/55 °C, 30 s/72 °C, 45 s
	Reverse	GGAAGGCTCTGGTTCAGCAA	95 °C, 30 s/55 °C, 30 s/72 °C, 45 s
Krüppel-like factor 4 (*Klf4*)	Forward	CAGACCAGATGCAGTCACAA	95 °C, 30 s/55 °C, 30 s/72 °C, 45 s
	Reverse	GTTTCTCGCCTGTGTGAGTT	95 °C, 30 s/55 °C, 30 s/72 °C, 45 s
Neurogenin 3 (*Neurog3*)	Forward	TCTCGCCTCTTCTGGCTTTC	95 °C, 30 s/55 °C, 30 s/72 °C, 45 s
	Reverse	AAGTCGGTGAAGAACGGACAA	95 °C, 30 s/55 °C, 30 s/72 °C, 45 s
SRY-Box Transcription Factor 9 (*Sox9*)	Forward	CAAGCACAACGGACCAGA	95 °C, 30 s/55 °C, 30 s/72 °C, 45 s
	Reverse	CAGCGCCTTGAAGATAGCATT	95 °C, 30 s/55 °C, 30 s/72 °C, 45 s
**GRP78 signaling pathway**			
Activating transcription factor 6 (*Atf6*)	Forward	CTGGGCTCGGTAGTTTGTATC	95 °C, 30 s/55 °C, 30 s/72 °C, 45 s
	Reverse	AGACCTGAATGGCTGCTTAC	95 °C, 30 s/55 °C, 30 s/72 °C, 45 s
X-box binding protein 1 (*Xbp1*)	Forward	CCTTCAGTGACATGTCTTCTCC	95 °C, 30 s/55 °C, 30 s/72 °C, 45 s
	Reverse	CCCAGTGTTATGTGGCTCTTTA	95 °C, 30 s/55 °C, 30 s/72 °C, 45 s
C/EBP homologous protein (*Chop*)	Forward	GGAGGTCCTGTCCTCAGATGAA	95 °C, 30 s/59 °C, 30 s/72 °C, 45 s
	Reverse	GCTCCTCTGTCAGCCAAGCTAG	95 °C, 30 s/59 °C, 30 s/72 °C, 45 s
Glycogen synthase kinase-3 beta (*Gsk3b*)	Forward	GAGCCACTGATTACACGTCCAG	95 °C, 30 s/59 °C, 30 s/72 °C, 45 s
	Reverse	CCAACTGATCCACACCACTGTC	95 °C, 30 s/59 °C, 30 s/72 °C, 45 s
Mammalian target of rapamycin (*mTor*)	Forward	AGAAGGGTCTCCAAGGACGACT	95 °C, 30 s/59 °C, 30 s/72 °C, 45 s
	Reverse	GCAGGACACAAAGGCAGCATTG	95 °C, 30 s/59 °C, 30 s/72 °C, 45 s

**Table 2:** List of antibodies.

Antibodies	Sources	Locations	Identifier Catalogue Numbers
Rat Ki-67 Monoclonal Antibody (SolA15)	Invitrogen	Winnipeg, MB, CA	14-5698-82
Rabbit Keratin 20 (D9Z1Z) XP^®^ antibody	Cell Signaling Technology	Danvers, MA, USA	13063S
Rabbit Anti-MUC2 (EPR23479-47) antibody	Abcam, Inc.	Waltham, MA, USA	ab272692
Rabbit Chromogranin A antibody	Invitrogen	Winnipeg, MB, CA	PA5-16685
Mouse Doublecortin (E-6) antibody	Santa Cruz	San Diego, CA, USA	sc-271390
Rabbit LGR5/GPR49 antibody	Bio-Techne	Minneapolis, MN, USA	NBP1-28904
Rat LY-6A/E (D7) antibody	Santa Cruz	San Diego, CA, USA	SC-52601
Mouse HOP (E-1) (HOPX) antibody	Santa Cruz	San Diego, CA, USA	SC-398703
Goat anti-Rat IgG (H+L) Cross-Adsorbed Secondary Antibody, Alexa Fluor™ 488	Invitrogen	Winnipeg, MB, CA	A11006
Goat anti-Rat IgG (H+L) Cross-Adsorbed Secondary Antibody, Alexa Fluor™ 647	Invitrogen	Winnipeg, MB, CA	A21247
Goat anti-Rabbit IgG (H+L) Highly Cross-Adsorbed Secondary Antibody, Alexa Fluor™ 488	Invitrogen	Winnipeg, MB, CA	A11034
Goat anti-Rabbit IgG (H+L) Cross-Adsorbed Secondary Antibody, Alexa Fluor™ 647	Invitrogen	Winnipeg, MB, CA	A21244
Goat anti-Mouse IgG (H+L) Cross-Adsorbed Secondary Antibody, Alexa Fluor™ 488	Invitrogen	Winnipeg, MB, CA	A11001

## Data Availability

The data that support the findings of this study are available from the corresponding author upon reasonable request.

## Supplementary Materials

The following supporting information can be downloaded at: https://www.mdpi.com/article/10.3390/ijms252312757/s1.
