# Supplementary material for: Pancreastatin Inhibition Alters the Colonic Epithelial Cells Profile in a Sex-Dependent Manner
Source: Int J Mol Sci. 2024 Nov 27;25(23):12757. doi: 10.3390/ijms252312757 (PMC11641590; doi:10.3390/ijms252312757)
Supplement: Supplementary file 1 [file ijms-25-12757-s001.zip › ijms-3238174-supplementary.pdf]

Pancreastatin Inhibition Alters the Colonic Epithelial Cells Profile in a Sex-Dependent Manner

Diane M. Tshikudi [1], Hannah Hutchinson [1], and Jean-Eric Ghia [1, 2, 3 *]

[1] Department of Immunology, Rady Faculty of Health Sciences, University of Manitoba, R3E 0T5
Winnipeg, MB, Canada; tshikudd@myumanitoba.ca (D.M.T.); hutchish@myumanitoba.ca (H.H.)

[2] Children's Hospital Research Institute of Manitoba, University of Manitoba, Winnipeg, MB, Canada, R3E 3P4

[3] IBD Clinical and Research Centre, University of Manitoba, Winnipeg, MB, Canada

***** Correspondence: jean-eric.ghia@umanitoba.ca (J.-E.G.); Tel.: +1-(204)-789-3802

[*]Correspondence: Jean-Eric Ghia, Ph.D., Professor
Immunology Department, University of Manitoba
Address: 750 McDermott Avenue, Winnipeg, Manitoba, R3E 0T5
Tel: +1 (204) 789-3802
E-mail: jean-eric.ghia@umanitoba.ca

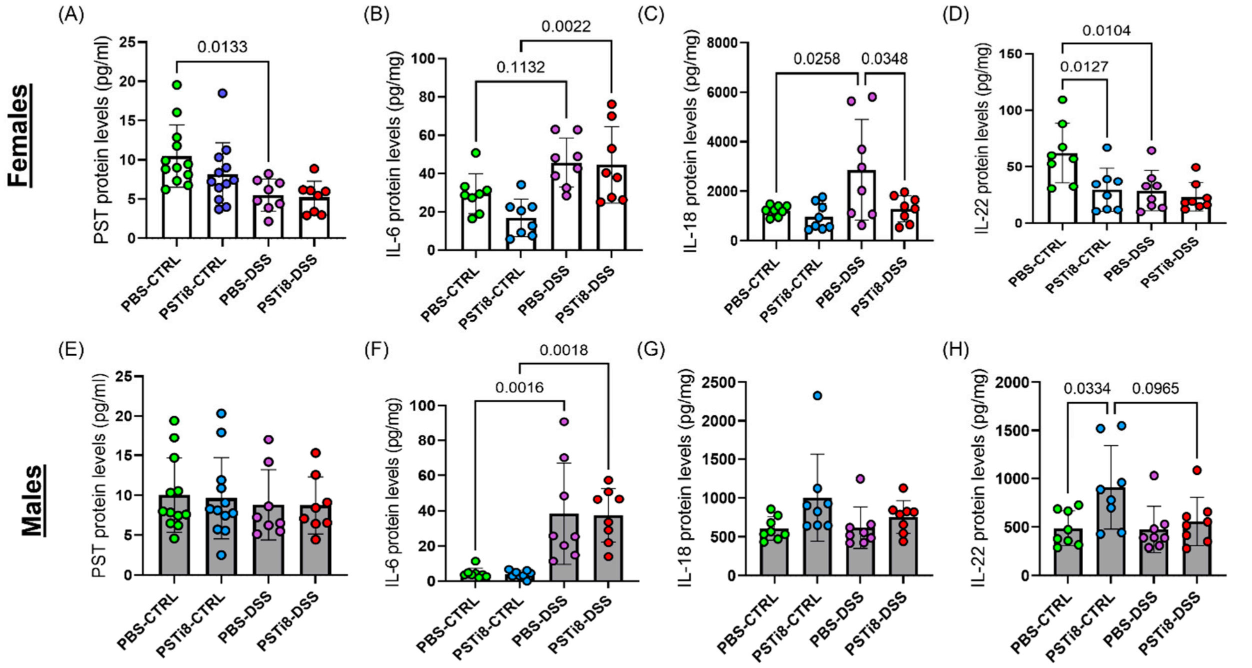

Figure S1. Effect of PSTi8-treatment on colonic mucosal cytokines profile in colitic compared to steady state conditions in male and female mice. Male and female C57BL/6 (WT) mice (n = 6-8) were administered with PSTi8 (2.5 mg/kg/day) or PBS by intra-rectal route one day before treatment with 5% DSS for five days to induce colitis (**A–G**). Colon tissues were collected and homogenized to extract proteins. Protein levels of PST (**A, E**) and mucosal regulatory inflammatory cytokines, Interleukin (IL)-6 (**B, F**), IL-18 (**C, G**), and IL-22 (**B–D** and **F–H**), were evaluated by ELISA. Data was expressed as the mean ± standard deviation. Statistical significance was determined using ordinary one-way ANOVA and Tukey's multiple comparisons test. Statistical analysis to determine outliers was performed using robust regression and outlier.

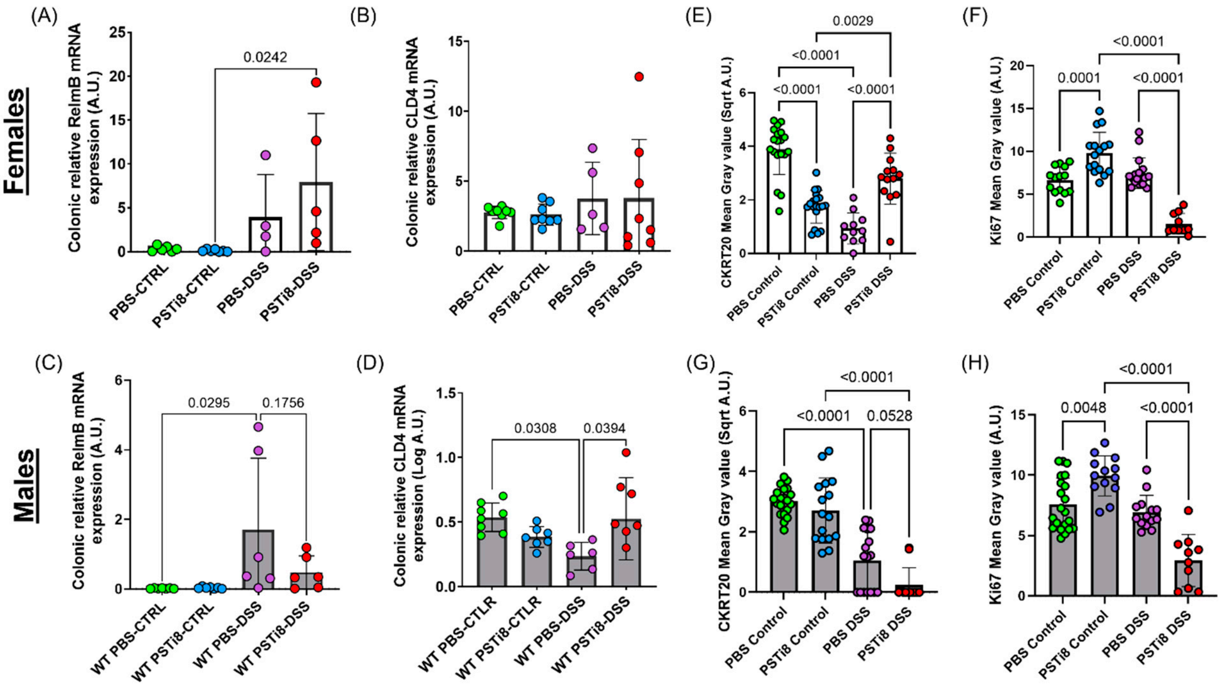

Figure S2. Effect of PSTi8-treatment on markers associated with the mucosal integrity in colitic compared to steady state conditions in male and female mice. (A-H) Colonic tissue collected from males and female C57BL/6 (WT) (n = 4–8) treated intrarectally with 2.5 mg/kg/day PSTi8 or PBS daily for 6 days concomitant with DSS or water for 5 days were used (control group, CTRL). mRNA expression of markers linked to microbiota mucosal colonization, *Relmβ*, and tight junction, claudin *(Cld)4*, were measured by qRT-PCR (**A–D**). Markers associated with epithelial differentiation (cytokeratin 20, CKRT20) and proliferation (KI67) were measured by immunofluorescence (**E–H**). Results represented the means ± standard deviation. One-way ANOVA followed by Tukey's post hoc was performed between groups. Statistical analysis to determine outliers was performed using robust regression and outlier.

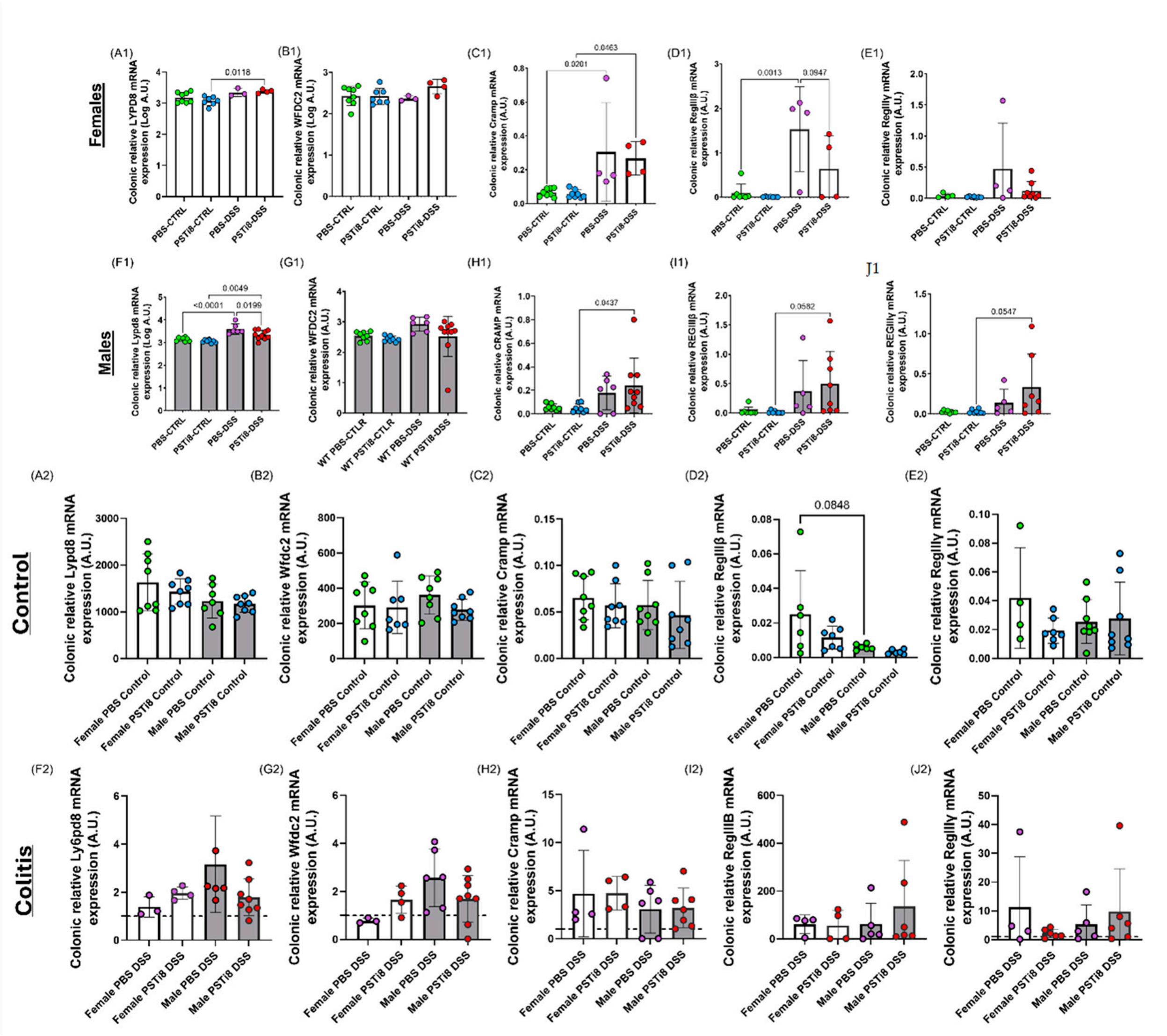

Figure S3. Effect of PST inhibition on colonic mucosal antimicrobial peptides expression in colitic compared to steady state conditions in male and female mice. Colonic tissue collected from males and female C57BL/6 (WT) (n = 4-8) treated intrarectally with 2.5 mg/kg/day PSTi8 or PBS daily for 6 days concomitant with DSS or water for 5 days were used (control group, CTRL) (**A1–J2**). mRNA expression of Ly6/Plaur domain containing 8 (*Lypd8*), WAP four-disulfide core domain protein (*Wfcd2*), cathelin-related antimicrobial peptide (*Cramp*), dysregulated expression of type III REG *(RegIII)γ* and *RegIIIβ* were measured by qRT-qPCR. Data from DSS-treated mice was normalized to control conditions (**F2–J2**). The dashed line in each figure represents a ratio of 1, indicating an absence of difference between DSS-induced colitis and control conditions. Results represented the means ± standard deviation. One-way ANOVA followed by Tukey's post hoc was performed between groups. Statistical analysis to determine outliers was performed using robust regression and outlier.

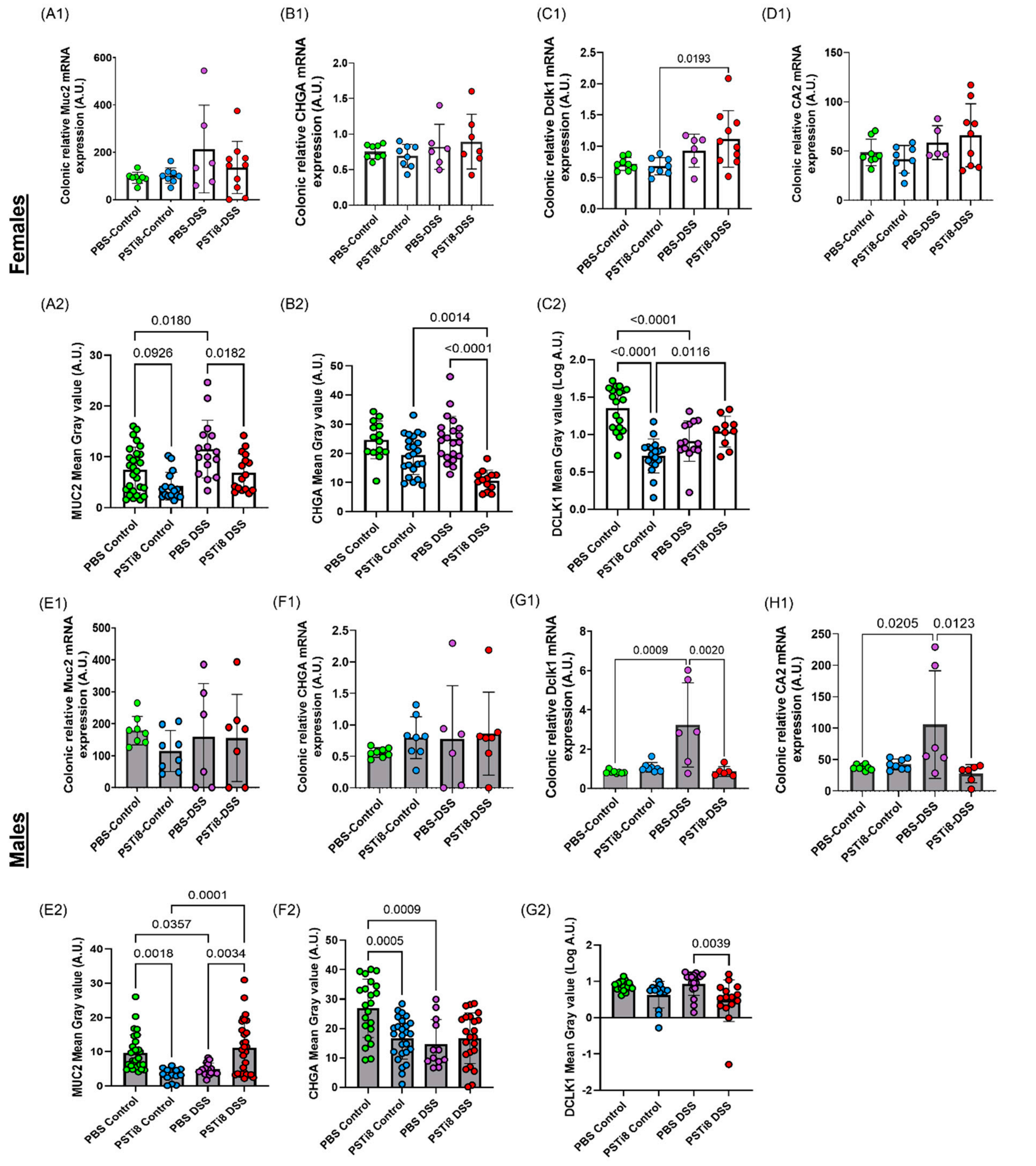

Figure S4. Effect of PST inhibition on terminally differentiated colonic epithelial cell markers in colitic compared to steady state conditions in male and female mice. Expression of markers specific to differentiated colonic epithelial cells was evaluated (**A1–G2**). Colonic tissues collected from males and female C57BL/6 (WT) (n = 5-8) treated intrarectally with 2.5 mg/kg/day PSTi8 or PBS daily for 6 days concomitant with DSS or water for 5 days were used. mRNA and protein levels of markers specific to goblet cells, mucin (MUC)2, enteroendocrine

cells, chromogranin A (CHGA), and Tuft cells, doublecortin-like kinase (DCLK) 1, were characterized by RT-qPCR (**A1–H1**) and immunofluorescence (IF) (**A2–G2**). Results represented the means ± standard deviation. One-way ANOVA followed by Tukey's post hoc was performed between groups. Statistical analysis evaluating using robust regression and outlier.

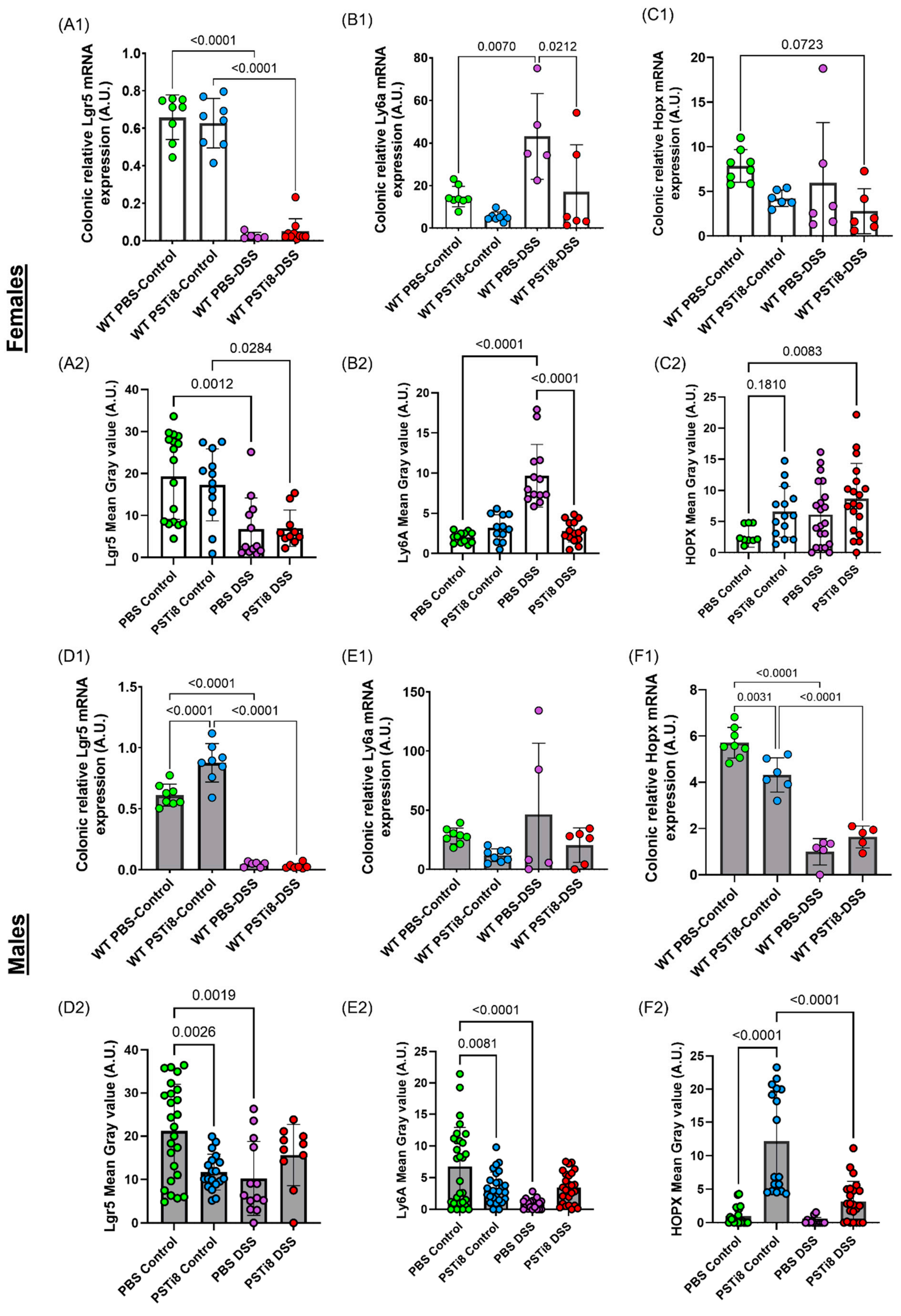

Females
(A1)
Colonic relative Lgr5 mRNA expression (A.U.)
<0.0001
<0.0001
WT PBS-Control
WT PSTi8-Control
WT PBS-DSS
WT PSTi8-DSS
(B1)
Colonic relative Ly6a mRNA expression (A.U.)
0.0070
0.0212
WT PBS-Control
WT PSTi8-Control
WT PBS-DSS
WT PSTi8-DSS
(C1)
Colonic relative Hopx mRNA expression (A.U.)
0.0723
WT PBS-Control
WT PSTi8-Control
WT PBS-DSS
WT PSTi8-DSS
(A2)
Lgr5 Mean Gray value (A.U.)
0.0012
0.0284
PBS Control
PSTi8 Control
PBS DSS
PSTi8 DSS
(B2)
Ly6A Mean Gray value (A.U.)
<0.0001
<0.0001
PBS Control
PSTi8 Control
PBS DSS
PSTi8 DSS
(C2)
HOPX Mean Gray value (A.U.)
0.0083
0.1810
PBS Control
PSTi8 Control
PBS DSS
PSTi8 DSS
Males
(D1)
Colonic relative Lgr5 mRNA expression (A.U.)
<0.0001
<0.0001
<0.0001
WT PBS-Control
WT PSTi8-Control
WT PBS-DSS
WT PSTi8-DSS
(E1)
Colonic relative Ly6a mRNA expression (A.U.)
WT PBS-Control
WT PSTi8-Control
WT PBS-DSS
WT PSTi8-DSS
(F1)
Colonic relative Hopx mRNA expression (A.U.)
<0.0001
0.0031
<0.0001
WT PBS-Control
WT PSTi8-Control
WT PBS-DSS
WT PSTi8-DSS
(D2)
Lgr5 Mean Gray value (A.U.)
0.0019
0.0026
PBS Control
PSTi8 Control
PBS DSS
PSTi8 DSS
(E2)
Ly6A Mean Gray value (A.U.)
<0.0001
0.0081
PBS Control
PSTi8 Control
PBS DSS
PSTi8 DSS
(F2)
HOPX Mean Gray value (A.U.)
<0.0001
<0.0001
PBS Control
PSTi8 Control
PBS DSS
PSTi8 DSS

Figure S5. Effect of PST inhibition on colonic stem cells markers in colitic compared to steady state conditions in male and female mice. The expression of markers associated with colonic epithelial stem cells was evaluated (**A1–F2**). Male and female C57BL/6 (WT) (n = 4-8 mice per group) were utilized for this study. Mice were treated with intrarectal injection with 2.5 mg/kg/day PSTi8 or PBS daily for 6 days. One day after starting PSTi8 treatment, mice received for 5 consecutive days 5% dextran sulfate sodium (DSS) added in the drinking water to induce colitis or with water for the control group (CTRL) Colon tissue extracted at day five post-DSS treatment was analyzed using RT-qPCR (**A1–F1**) and immunofluorescence (**A2–F2**) (n=3). Biomarkers specific to crypt base columnar (LGR5), paligenosis (fetal-like) (LY6A), and damage-associated regenerative (HOPX), colonic stem cells were evaluated. Histogram data represent the mean ± standard deviation. One-way ANOVA followed by Tukey's post hoc was performed between groups. Statistical analysis to determine outliers was performed using robust regression and outlier.

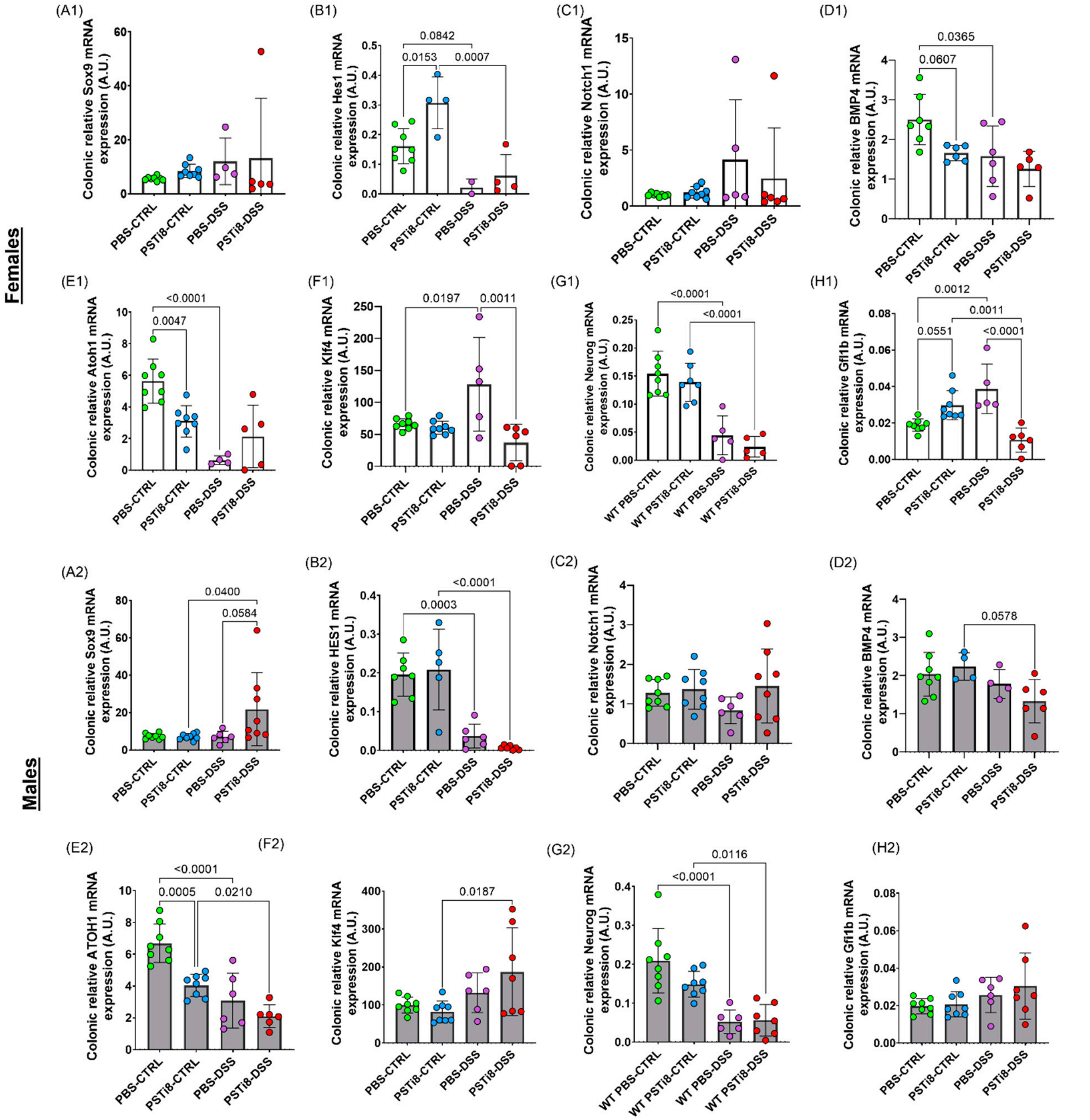

Figure S6. Effect of PST inhibition on colonic epithelial cell lineage commitment markers in colitic compared to steady state conditions in male and female mice. Males and females C57BL/6 (WT) (n = 4-8) were administered with PSTi8 (2.5 mg/kg/day) or PBS by intra-rectal route one day before treatment with 5% DSS to induce colitis. mRNA expression of markers specific for transcription factors mediating stem cell renewal and differentiation *Sox9*, *Hes1*, absorptive progenitors (*Notch1*), and *Bmp4*, secretory progenitors (*Atoh1*), along with differentiation of goblet cells (*Klf4*), enteroendocrine *Neurog*., and Tuft cells, (*Gfi1b*) were measured by RT-qPCR (**A1–H2**). Histogram data represent the mean ± standard deviation. One-way and two-way ANOVA followed by a multiple comparison test, were assessed. One-way

ANOVA followed by Tukey's post hoc was performed between groups. Statistical analysis to determine outliers was performed using Robust regression and Outlier.

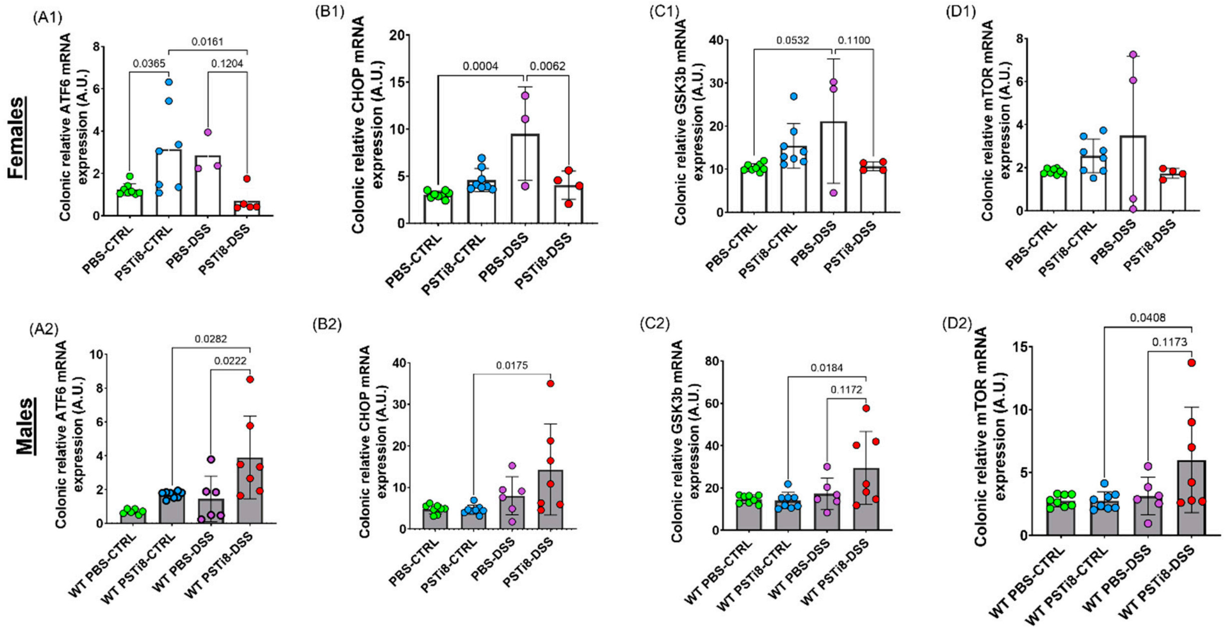

Figure S7. Effect of PST inhibition on mucosal endoplasmic reticulum (ER) stress, mediators of apoptosis, and proliferation markers in colitic compared to steady state conditions in male and female mice. Colon tissue from males and females C57BL/6 (WT) (n = 3-8) administered with PSTi8 (2.5 mg/kg/day) or PBS by intra-rectal route one day before treatment with 5% DSS to induce colitis will be used. Biomarkers specific to ER stress (*Atf6, Xbp1*) (**A1–B2**), apoptosis (*Chop*) (**C1–C2**), and inhibition of cell growth (*mTor, Gsk3β*) (**D1–E2**) were assessed from colon samples by RT-qPCR. Histogram Data represent the mean ± standard deviation. One-way ANOVA followed by a multiple comparison test, was performed. One-way ANOVA followed by Tukey's post hoc was performed between groups. Statistical analysis to determine outliers was performed using robust regression and outlier.
